# Personality traits and the managerial capacity of community-based facilities providing HIV services to key populations in Kenya and Malawi

**DOI:** 10.1371/journal.pone.0352752

**Published:** 2026-06-26

**Authors:** Andrea Salas-Ortiz, Marjorie Opuni, José Luis Figueroa, Louis Masankha Banda, Alice Olawo, Spy Munthali, Julius Korir, Barbara Nyambura Thirikwa, Agatha Bula, Navindra Persaud, Sergio A. Bautista-Arredondo

**Affiliations:** 1 Centre for Health Economics, University of York, York, United Kingdom; 2 Independent Consultant, Geneva, Switzerland; 3 Center for Evaluation Research and Surveys (CIEE), National Institute of Public Health (INSP), Cuernavaca, Mexico; 4 FHI 360, Lilongwe, Malawi; 5 FHI 360, Nairobi, Kenya; 6 University of Malawi, Zomba, Malawi; 7 Kenyatta University, Nairobi, Kenya; 8 Independent Consultant, Nairobi, Kenya; 9 UNC Project, Lilongwe, Malawi; 10 FHI 360, Washington, District of Columbia, United States of America; 11 Economic Analysis Unit, Ministry of Health, Mexico City, Mexico; University of Ghana College of Health Sciences, GHANA

## Abstract

Community-based facilities delivering HIV services to key populations often face significant operational and financial challenges, making the quality of management in these organizations particularly important. A growing body of evidence links management quality to health facility performance. However, research on management in health facilities has focused largely on structural characteristics and formal qualifications, with less attention to the non-cognitive characteristics of managers themselves, particularly personality traits. This is a cross-sectional, quantitative analysis of 45 facilities providing HIV services to key populations in Kenya and Malawi. The analysis includes two stages. We first use k-means cluster analysis on management practice data to identify sub-groups of facilities that share similar management profiles. We then use non-linear logit regression models to predict the probability of facilities belonging to each sub-group as a function of manager personality traits, controlling for manager education, experience, and facility location characteristics. We used the Big Five Inventory to measure personality traits. We found two clusters of facilities with statistically different levels of managerial capacity. The higher-capacity cluster shows stronger financial and people management, more developed performance monitoring and target setting, better operations management, and greater community involvement in financial decisions. In the non-linear logit models, educational achievement was not statistically significant. Manager experience working with HIV key populations and higher scores on the Stability meta-trait were positively and significantly associated with the probability of belonging to the high-managerial capacity cluster. The personality traits of managers, together with their technical and cognitive skills, are relevant to the selection and support of managers in these organizations.

## Introduction

HIV services for key populations—men who have sex with men, female sex workers, people who inject drugs, and transgender women—tend to be most effective when delivered through community-based approaches that enhance their accessibility and acceptability [1,2]. However, the community-based organizations delivering these services often face significant operational challenges. These organizations provide a complex package of services, including clinical care and non-clinical interventions such as empowerment activities, stigma reduction programs, education, and peer outreach [3–5], delivered at fixed sites and directly within communities [6,7]. These organizations also operate under significant human resource constraints, relying on diverse service providers, temporary workers, and community volunteers who need management support, technical guidance, training, and oversight from higher-level institutions [8]. Many of these organizations also face financing challenges, often depending on multiple funding sources, each with its own performance monitoring and reporting requirements [9,10].

Given these challenges, the role of effective management may be particularly important in community-based facilities providing HIV services to key populations. A growing body of evidence links management quality to health facility performance [11–13]. Studies have shown that more effective management is associated with superior clinical outcomes [14,15], better quality of care [12,15–21], and higher levels of efficiency, including in HIV services [16,20,22,23]. A study of voluntary medical male circumcision, however, found no statistically significant relationship between management practices and efficiency [24].

Effective management is reflected in the implementation of good management practices, including target setting, performance monitoring, people management, operations management, financial management, and community engagement [11,25–29]. Our previous research in Kenya and Malawi documented considerable variation in the management practices of community-based HIV facilities, with some exhibiting high levels of management competence and others low levels [30]. In that study, we adjusted management scores for facility characteristics, operational experience, location, manager education, and proximity to comparable sites, but substantial variation in scores remained that these characteristics did not explain [30]. Effective management also depends on manager behavior, and research suggests that the behavior of managers shapes organizational performance, both in the private sector [31] and in public health systems [32]. Personality traits are among the manager characteristics that may matter. They have been examined more in relation to leadership effectiveness [33] than in relation to management capacity in health facilities.

Personality traits are patterns of thinking, feeling, and behaving that are distinct from cognitive ability and educational attainment [34,35]. Evidence shows that these traits are associated with a variety of social and economic outcomes [34,36–38], although according to Trait Activation Theory, their relevance in any given role depends on the demands of the work [39]. For managers of community-based facilities providing HIV services to key populations, these demands include coordinating the delivery of complex services, managing diverse staff, handling multiple funding streams, and responding to the needs of communities that continue to face significant stigma and legal barriers to care.

This paper examines the association between manager personality traits and managerial capacity in community-based facilities providing HIV services to key populations as part of the Linkages Across the Continuum of HIV Services for Key Populations Affected by HIV (LINKAGES) program in Kenya and Malawi. Services were delivered by local organizations called implementing partners (IPs) in facilities called drop-in centers (DICs) and in communities. We investigate the relationship between managerial capacity and the Big Five personality traits—Openness/Intellect, Conscientiousness, Extraversion, Agreeableness, and Emotional Stability—as well as the higher-order meta-traits of Stability (which aggregates Conscientiousness, Agreeableness, and Emotional Stability) and Plasticity (which aggregates Openness/Intellect and Extraversion) [40–44]. We aim to contribute to the growing literature on health management in low-resource settings and provide insights to inform recruitment, training, and support strategies for community-based facility managers.

## Methods

### Study design

This study employs a cross-sectional, quantitative design to examine the relationship between manager personality traits and management practices in DICs. We utilize a multi-stage analytical approach that combines K-means clustering to identify management profiles and non-linear econometric techniques to model the probability of DICs belonging to these groups based on manager personality traits, controlling for education and experience.

### Study Setting

This study analyzes data from the LINKAGES program in Kenya and Malawi, funded by the United States Agency for International Development (USAID) through the United States President’s Emergency Plan for AIDS Relief (PEPFAR) and administered by FHI 360 from 2014 to 2021. The program, implemented in Africa, Asia, and the Americas, aimed to reduce HIV transmission among key populations and improve their enrollment and retention in care and treatment. LINKAGES delivered a comprehensive package of HIV services to key populations, including female sex workers, men who have sex with men, transgender people, and people who inject drugs.

A central pillar of the LINKAGES service delivery model was the establishment of DICs, which are community-based health facilities designed to address the needs of KPs who often face significant stigma, discrimination, and legal barriers at traditional health facilities. DICs are frequently KP-led, ensuring a non-judgmental environment that fosters trust and consistent engagement with the healthcare system.

Clinical interventions provided in these centers include HIV testing services, antiretroviral therapy, post-exposure prophylaxis, pre-exposure prophylaxis, sexually transmitted infection treatment, sexual and reproductive health services, and the management of sexual violence. Alongside these clinical services, LINKAGES also provided non-clinical services such as empowerment programs and community engagement initiatives. LINKAGES also included pre-service activities such as population mapping and size estimation, as well as management and monitoring efforts at higher levels. Local community-based and key population-led organizations delivered HIV services at DICs and in communities, overseen by LINKAGES country offices and headquarters in the United States. A comprehensive overview of the LINKAGES program can be found elsewhere [45,46].

### Sampling and sample size

The study utilized a purposive sampling strategy to include DICs under the LINKAGES program in Kenya and Malawi. A total of 44 DIC managers were identified and invited to participate (29 from Kenya and 15 from Malawi), achieving a 100% response rate.

While 44 managers participated, the final analytical sample consists of 45 observations. This is because one manager in Kenya was responsible for overseeing two distinct DICs with separate organizational structures and performance data. Therefore, management practices were assessed for both sites. The final distribution reflects 67% of facilities in Kenya and 33% in Malawi. All included DICs provided HIV services to key populations, ensuring a comparable sampling frame across both national contexts.

### Data collection

The data-collection instrument for this study has been described in detail elsewhere [30]. The survey tool was developed in collaboration with LINKAGES headquarters and the LINKAGES country offices in Kenya and Malawi. Data on DIC characteristics, management practices, and manager personality traits were collected through an online survey administered to DIC managers. The survey was applied from 1 November 2021–31 January 2022 using the SurveyMonkey Inc. (San Mateo, California, USA; Main Website: www.surveymonkey.com) platform.

## Measurement of variables

### Dependent variable: Management clusters

The dependent variable in our analysis is a binary indicator representing a DIC’s belonging to a *lower* or *higher* management cluster. This membership could be identified by splitting a management score at some point of its distribution (e.g., at the mean or median). However, this would likely lack conceptual meaning in skewed distributions. Thus, to identify distinct groups of DICs based on multiple management characteristics simultaneously, we used the data-driven method of k-means clustering, an unsupervised machine-learning method. This method partitions observations into a pre-specified number of non-overlapping clusters, where observations within each cluster share similar characteristics, while observations in different clusters do not, better identifying management profiles.

This approach was chosen for three primary reasons. First, clustering treats management as a multidimensional construct, capturing potential complementarity between different management dimensions. Second, we used eight specific management-related variables (Table A3) as inputs for the clustering that focus on managerial agency, rather than procedural compliance. Third, although we constructed broader management scores, these eight variables were excluded from these indicators. This allowed us to perform an internal validity check (see further details in the Management Practice Scores subsection).

**Fig 1:**
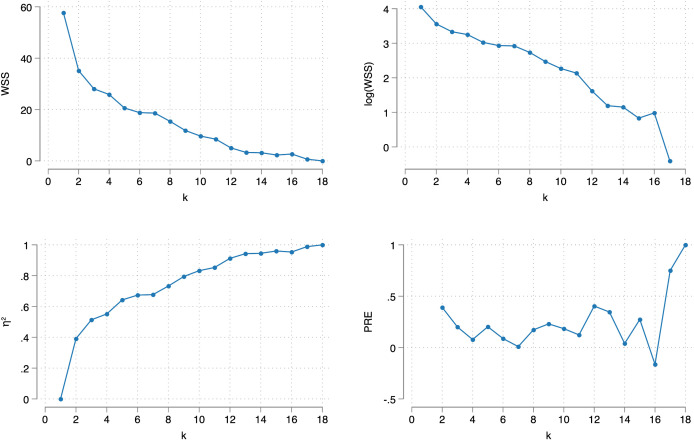
WSS, log (WSS), $\eta^{2}$, and PRE for all *k* cluster solutions.

Since the number of clusters is chosen arbitrarily by the algorithm, we determined the optimal number of clusters following the approach proposed by Makles (2012) [47]. This method consists of identifying several *k*-means solutions with different numbers of clusters and comparing them using within-cluster sum of squares (WSS) and proportional reduction of error coefficient [47]. The *k*-means algorithm assigns each observation to a *k* cluster to minimize within-cluster variation, summed over all clusters. Our analysis identified two optimal DIC clusters as evidenced by the noticeable kink in the WSS at *k* = 2 (Figure 1). Furthermore, $\eta^{\text{2}}$ points to a reduction of the WSS by approximately 40% at *k* = 2 and minimal additional reduction for *k* > 2.

### Characterization of clusters: Management practice scores

To provide substantive meaning to these clusters, we used a separate set of management practices scores as an independent validation check. These scores were constructed following the World Management Survey (WMS) framework [25] which focuses on four domains: target setting (establishing appropriate targets, tracking relevant outcomes, and ensuring alignment between targets and outcomes); performance monitoring (collecting and analyzing data to understand organizational performance and identify improvement opportunities); people management (activities related to hiring, retaining, and rewarding high performance, and addressing underperformance), and operations management (standardizing processes and continuously improving organizational operations) [25,26,29]. Furthermore, two additional management domains were included in this low-resource context: financial management (budgeting and financial accounting of revenues and expenses), and community engagement (developing and managing relationships with community members through community outreach, involvement of community leaders, and activities to ensure customer trust and satisfaction) [11,27,28]. A list of the items included in each management domain is provided in Table A1.

Scores for each management dimension were calculated by aggregating the items in Table A1. We calculated seven management scores for each DIC: six for the individual dimensions and an aggregated management score consolidating overall management capacity into a single indicator. We then characterize the clusters by comparing the groups based on the distribution of the overall management score, the six management dimensions that comprise this score, and selected DIC and manager characteristics. This allowed us to label the clusters as *lower* and *higher* management levels based on statistically significant differences in organizational practices.

Table 1 shows the differences in management and contextual characteristics between the two groups. We found statistically significant mean differences in the general management score and most management dimensions, except for performance monitoring and target setting. The groups were statistically similar in terms of DIC location and manager age and sex.

**Table 1:** Management scores and selected characteristics by DIC management cluster.

DIC and manager characteristics	*Lower* management level					*Higher* management level					Mean diff. p-value
	N	Mean	Median	Min	Max	N	Mean	Median	Min	Max	
***Management (continuous variables)***											
**General score**	29	62.55	55.87	10.93	90	16	78.65	72	10.37	96.67	0.000
**Community involvement**	29	44.83	50.00	0.00	75.00	16	87.50	100.00	25.00	100.00	0.000
**Financial management**	29	34.48	0.00	0.00	100.00	16	61.71	87.51	0.00	100.00	0.044
**Operations management**	29	93.10	100.00	72.73	100.00	16	97.73	100.00	90.91	100.00	0.082
**People management**	29	30.69	30.00	10.00	65.00	16	43.13	42.50	20.00	80.00	0.008
**Performance monitoring**	29	79.12	83.33	44.44	100.00	16	85.42	91.67	50.00	100.00	0.237
**Target setting**	29	93.10	100.00	57.14	100.00	16	96.43	100.00	71.43	100.00	0.282
***DIC characteristics and manager’s demographics (binary variables)***											
***DIC location: Country***											
Malawi	11	37.9%	–	–	–	4	25.0%	–	–	–	0.294
Kenya	18	62.1%	–	–	–	12	75.0%	–	–	–	
***IP management type***											
DICs from IPs with a unique DIC	5	17.2%	–	–	–	8	50.0%	–	–	–	0.025
DICs from IPs with two or more DICs	24	82.8%	–	–	–	8	50.0%	–	–	–	
***Manager age group***											
Young adults	16	55.2%	–	–	–	6	37.5%	–	–	–	0.205
Middle-aged adults	13	44.8%	–	–	–	10	62.5%	–	–	–	
***Manager sex***											
Male	13	44.8%	–	–	–	6	37.5%	–	–	–	0.438
Female	16	55.2%	–	–	–	10	62.5%	–	–	–	

Note: Diff. means difference.

This approach serves as an internal validity test because the clustering method naturally identifies the groups that exhibit statistically significant differences in the independent management practices scores. This provides objective empirical evidence that the clusters represent distinct, high/low management profiles rather than a predetermined or hand-picked mechanical artifact.

### Main independent variable: Personality traits

Manager personality traits were measured using the 15-item Big Five personality inventory—an abbreviated version of the most prominent measurement system for personality traits [40,48]. This inventory assesses five personality traits using three items each (Table A2 shows all the items included to create each personality trait). Openness/Intellect (intelligent, original, curious, and ingenious); Conscientiousness (orderly, responsible, dependable, and hard-working); Extraversion (talkative, assertive, and sociable); Agreeableness (cooperative, unselfish, good-natured, trustworthy, and friendly); and Emotional Stability (predictable and consistent emotional reactions, even when facing challenges) [35,41,42,49]. It represents the positive side of what was originally termed Neuroticism (anxiety, vulnerability, irritability). More recent studies have shown that these five traits consistently relate to each other in a way that indicates two meta-personality traits: Stability (comprising Conscientiousness, Agreeableness, and Emotional Stability) and Plasticity (comprising Openness/Intellect and Extraversion) [43,44,50]. While Stability involves the disposition to cope with stress, Plasticity involves the disposition towards exploration, flexibility, and adaptation to novel situations [51].

To address potential measurement error and cultural noise in self-reported data, we constructed aggregate scores for each personality and meta-personality trait following Anderson’s procedure [52]. By calculating standardized weighted average z-scores based on the inverse of the covariance matrix, this approach statistically prioritizes items with higher informational content and down-weights redundant items. This cleans the data so that cross-cultural variations do not distort the measurement of the trait, ensuring scores are robust and provide a more precise estimation of the underlying latent traits than a simple linear aggregation.

### Econometric analysis

To examine the relationship between manager personality traits and the probability of a DIC belonging to the higher management cluster, we employed a series of non-linear logit models. We adopted a stepwise modeling approach across eight specifications to test the stability of our estimates.

The probability of a DIC *k* overseen by manager *i* belonging to the higher managerial capacity group ($Y_{ij}=\text{1}$) is estimated using the following model:

$P(Y_{ik}=\text{1}|E_{i},P_{i},\;X_{ik})=f(\alpha +\beta E_{i}+\delta P_{i}+\gamma X_{ik}+\epsilon_{ik})$

Where *E* represents a vector of manager formal education and accumulated experience. *P* represents manager personality traits, and *X* is a vector of facility and demographic controls. The eight model specifications are defined by the sequential inclusion of these vectors. Model I establishes a baseline, estimating the probability of cluster membership as a function of manager formal education and years of experience working with key populations. Model II introduces our primary variables of interest: the personality meta-traits Stability and Plasticity. To test whether personality serves as a proxy for other factors, we conditioned the baseline model on a sequence of specific covariates. Model III adds country fixed effects to account for national-level cultural or policy differences, while Model IV controls for organizational structure by distinguishing between managers overseen by larger IP versus those in unique facilities. Models V and VI include manager age (young vs middle-aged) and sex, respectively, to ensure the trait effects are not confounded by demographic characteristics. Finally, Models VII and VIII present saturated specifications. Model VII includes the full suite of controls, while Model VIII provides a parsimonious robust specification. In all models, standard errors are clustered at the DIC level to account for potential intra-site correlation.

We report results using two complementary metrics. We show odds ratios, which provide the relative likelihood of a DIC belonging to the higher management cluster. However, because odds ratios can be difficult to interpret in terms of absolute impact, we also report average marginal effects for personality traits. This provides a more robust and intuitive estimate of the actual percentage-point shift in the probability of cluster membership associated with a unit increase in each personality trait.

### Ethical clearance

The study received ethical approval from the ethical review board of the National Institute of Public Health of Mexico (Number: 1554), the Kenya Medical Research Institute, and the National Commission for Science, Technology, and Innovation (Protocol No. 4258), and the National Commission on Research Ethics in the Social Sciences and Humanities of Malawi (Protocol No. P/07/21/590). All DIC managers who participated in the management survey completed an electronic informed consent form. Throughout the analysis, the authors did not have access to information that could identify individual participants.

## Results

As shown in Table 2, managers in the higher management cluster showed higher levels of Conscientiousness, Extraversion, and Emotional Stability compared to those in the lower managerial capacity cluster, with these differences being statistically significant. Average levels of Openness/Intellect and Agreeableness did not differ significantly between clusters. However, when examining the two personality meta-traits, managers in the higher management cluster had, on average, higher levels of Plasticity and Stability than managers in the lower-level group. In contrast, neither the level of manager schooling nor years of experience showed significant differences between clusters, suggesting that personality traits may be a more sensitive differentiator of managerial capacity groups in this context than traditional human capital metrics alone.

**Table 2:** Description of DIC clusters by manager personality traits and other characteristics.

Variables included	*Lower* management level					*Higher* management level					Mean diff. p-value
**N**	**Mean**	**SD**	**Min**	**Max**	**N**	**Mean**	**SD**	**Min**	**Max**
**Manager personality traits** ^**a**^											
*Big Five personality traits*											
Openness/Intellect	29	−0.22	1.04	−2.76	1.32	16	−0.04	1.14	−2.69	1.32	0.6098
Conscientiousness	29	−0.22	0.94	−2.86	0.98	16	0.37	0.70	−0.94	0.98	0.0323
Extraversion	29	−0.34	0.97	−2.32	1.70	16	0.43	0.85	−1.11	1.70	0.0106
Agreeableness	29	−0.10	1.12	−3.10	1.20	16	0.36	0.88	−1.11	1.20	0.1631
Emotional stability	29	−0.26	1.16	−3.10	1.19	16	0.46	0.72	−1.20	1.19	0.0293
*Personality meta-traits*											
Plasticity	29	−0.19	0.52	−1.24	0.73	16	0.12	0.52	−0.79	0.95	0.0562
Stability	29	−0.15	0.69	−1.71	0.96	16	0.33	0.54	−0.56	0.96	0.0206
**Control variables**											
*Level of manager schooling*											0.509
High school, nursery school, some college credit, technical training, or associate degree	14	48.3%	–	–	–	7	43.8%	–	–	–	
Bachelor’s degree or Postgraduate degree	15	51.7%	–	–	–	9	56.3%	–	–	–	
*DIC manager experience*											
Number of years of experience working with HIV Key Populations	29	5.9	5	2	11	16	6.75	6	4	9	0.2586

Notes: ^a^Expressed in z-scores. Diff. means difference.

Table 3 presents the results from the non-linear logit models focusing on the two meta-traits. Across all specifications, both meta-personality traits— Stability and Plasticity—were positively associated with the probability of belonging to the higher management cluster. However, Stability emerged as a consistent predictor of belonging to the higher managerial capacity cluster. In the core specification (Model III), a one standard deviation increase in Stability was associated with a 4.9-fold increase in the odds of belonging to the higher management cluster. In terms of absolute probability, the average marginal effect indicates that a one-unit increase in Stability is associated with a 26.5 percentage point increase in the probability of a DIC being in the high managerial capacity group.

**Table 3:** Results from the non-linear logit models using two meta-traits.

	Model specifications	I	II	III	IV	V	VI	VII	VIII
	**Variables included**	***Dependent variable: higher management = 1***							
**Meta-personality traits (odd ratios)**	Plasticity		2.345	2.403	2.841	2.322	2.413	3.676	3.686
			(2.554)	(2.517)	(3.342)	(2.536)	(2.759)	(4.494)	(4.526)
	Stability		4.850**	4.961**	4.217*	4.757**	4.544*	4.183	4.766*
			(3.623)	(3.847)	(3.575)	(3.717)	(3.806)	(4.154)	(4.081)
**Controls**	Highest degree or level of schooling you have completed: bachelor’s degree or Postgraduate degree	1.050	1.267	1.245	1.625	1.305	1.336	1.679	1.487
		(0.586)	(0.804)	(0.745)	(1.117)	(1.032)	(0.945)	(1.450)	(1.033)
	Manager years of experience^a^	2.065**	3.884***	3.979***	3.519***	3.736***	3.928***	4.163***	4.023***
		(0.747)	(1.510)	(1.369)	(1.136)	(1.467)	(1.461)	(1.365)	(1.338)
	Kenya (Ref: Malawi)			0.851				0.308	0.307
				(0.822)				(0.366)	(0.325)
	DICs from IPs with two or more DICs (Ref: DICs from IPs with a unique DIC)				0.233*			0.124**	0.134**
					(0.198)			(0.122)	(0.132)
	Middle-aged adults (Ref: Young adults)					1.153		0.978	
						(0.973)		(0.992)	
	Female (Ref: Male)						1.282	1.703	
							(1.013)	(1.332)	
**Average Marginal Effects**	Plasticity		0.141	0.145	0.158	0.139	0.145	0.186	0.190
	Stability		0.261**	0.265**	0.218*	0.257*	0.249*	0.205	0.227*
	Observations	45	45	45	45	45	45	45	45
	Pseudo R-squared	0.052	0.238	0.238	0.292	0.238	0.239	0.321	0.315

Notes: Coefficients expressed in odds ratios. Standard errors in parentheses. *** p < 0.01, ** p < 0.05, * p < 0.1. ^a^Expressed in z-scores. Average Marginal Effects represent the mean of the marginal effects calculated for each observation in the sample.

The manager’s years of experience were the most robust predictor. Although schooling was positively associated with the outcome, it did not reach statistical significance in any of the multivariate models, suggesting that specialized experience is a more critical driver of managerial capacity than general academic knowledge in these settings.

The analysis of individual traits (Table 4) provides further nuance to the meta-trait findings. Within the Stability domain, Conscientiousness showed a consistent positive association with higher managerial capacity across most of the specifications. In the core Model III, for instance, a one-standard-deviation increase in Conscientiousness was associated with an 18.1 percentage point increase in the probability of being in the higher management cluster (average marginal effect). While the smaller sample size warrants a cautious interpretation of individual predictors, Extraversion also trended positively. Overall, while the small number of observations limits our ability to make definitive claims about every trait, the directional consistency of the personality coefficients across all models suggests a robust underlying relationship between manager experience and managerial capacity.

**Table 4:** Results from the non-linear logit models using the Big Five inventory scores.

	Model specifications	II	III	IV	V	VI	VII	VIII
	**Variables included**	***Dependent variable: higher management = 1***						
**Personality traits** **(odd ratios)**	Openness/Intellect	0.929	0.921	1.045	0.924	0.936	1.059	1.070
		(0.319)	(0.308)	(0.431)	(0.301)	(0.329)	(0.398)	(0.502)
	Conscientiousness	3.147**	3.306**	2.489*	3.089**	3.010*	2.580	2.775
		(1.691)	(1.900)	(1.311)	(1.777)	(1.695)	(1.870)	(1.809)
	Extraversion	2.053	2.198	2.007	2.056	2.115	2.597	2.583
		(1.254)	(1.250)	(1.321)	(1.259)	(1.342)	(1.703)	(1.596)
	Agreeableness	1.116	1.146	1.072	1.113	1.067	1.128	1.214
		(0.255)	(0.274)	(0.278)	(0.259)	(0.318)	(0.377)	(0.373)
	Emotional stability	1.356	1.342	1.749	1.359	1.350	1.787	1.844
		(0.373)	(0.378)	(0.687)	(0.368)	(0.364)	(0.732)	(0.763)
**Controls**	Highest degree or level of schooling you have completed: bachelor’s degree or Postgraduate degree	1.434	1.394	1.895	1.475	1.585	2.059	1.649
		(0.917)	(0.872)	(1.325)	(1.118)	(1.123)	(1.906)	(1.102)
	Manager years of experience ^a^	3.411***	3.690***	2.739***	3.278***	3.456***	3.313***	3.290***
		(1.374)	(1.543)	(0.862)	(1.339)	(1.379)	(1.042)	(1.158)
	Kenya (Ref: Malawi)		0.665				0.204	0.204
			(0.656)				(0.230)	(0.212)
	DICs from IPs with two or more DICs (Ref: DICs from IPs with a unique DIC)			0.179			0.080***	0.085***
				(0.188)			(0.078)	(0.080)
	Middle-aged adults (Ref: Young adults)				1.144		1.020	
					(1.008)		(1.011)	
	Female (Ref: Male)					1.506	1.930	
						(1.448)	(2.042)	
**Average Marginal Effects**	Openness/Intellect	−0.011	−0.012	0.006	−0.012	−0.010	0.007	0.009
	Conscientiousness	0.173**	0.181**	0.124*	0.170**	0.165**	0.120	0.131
	Extraversion	0.109	0.119*	0.095	0.109	0.112	0.121*	0.122*
	Agreeableness	0.017	0.021	0.010	0.016	0.010	0.015	0.025
	Emotional stability	0.046	0.044	0.076	0.046	0.045	0.073	0.079
	Observations	45	45	45	45	45	45	45
	Pseudo R-squared	0.290	0.294	0.349	0.291	0.294	0.390	0.383

Notes: Coefficients expressed in odds ratios. Standard errors in parentheses. *** p < 0.01, ** p < 0.05, * p < 0.1. ^a^Expressed in z-scores. Average Marginal Effects represent the mean of the marginal effects calculated for each observation in the sample.

## Discussion

This study examines the relationship between manager personality traits and management capacity in 45 community-based facilities providing HIV services to key populations in Kenya and Malawi; we identified two distinct clusters of facilities with lower and higher management levels. We also found certain manager personality traits to be correlated with managerial capacity, suggesting that these traits may be important factors that have been overlooked when studying health units’ performance.

Overall, our results are consistent with previous research suggesting the need to extend human resource training beyond enhancing knowledge and skills to build non-cognitive and socio-emotional skills, to enhance attitudes and shift norms [53]. A key study finding was the strong association between manager Stability scores and management level, with facilities managed by managers with higher Stability scores almost five times more likely to be in the higher management cluster.

Our findings also suggest that the Stability meta-trait is more strongly associated with managerial capacity than Plasticity in the facilities studied. Consistent with Trait Activation Theory, we hypothesize that Stability, which is associated with maintaining consistency and structure [39, 43], may be particularly relevant in facilities with limited resources that deliver complex services to marginalized clients, where managers manage diverse staff with varying qualifications. In contrast, Plasticity, which reflects openness to new information [43] and creativity [51], may matter less in the LINKAGES context, where significant above-facility oversight was provided by LINKAGES country offices and headquarters, likely streamlining new information for DIC managers. For example, delivery of health services by LINKAGES takes more of a project approach than would be the case in a standard or routine government facility, where managers are encouraged to be innovative/creative to cover for shortages in funding for activities. We did not assess the intensity of above-facility support directly, and there may have been variation across facilities.

Among the Big Five personality traits, while Openness/Intellect, Conscientiousness, Extraversion, Agreeableness, and Emotional Stability were all positively associated with management level, only the association with Conscientiousness was statistically significant. This finding is consistent with previous research showing that Conscientiousness is a predictor of various economic outcomes [35,54–56]. Conscientious individuals, characterized by their organization, responsibility, and achievement orientation, may be more likely to implement essential management practices, such as systematic monitoring, consistent staff supervision, and sound financial controls.

Our findings suggest several potential implications for the human resource management of community-based facilities providing HIV services to key populations. The associations we found between personality traits and management levels indicate that organizations might consider incorporating some assessment of personality traits when selecting potential managers [57]. Our findings also suggest it could be valuable to consider these characteristics in leadership development training and the design of support programs for these managers. Research shows that personality traits are malleable and can be developed throughout life [58,59]. The extent to which interventions to develop these personalities could improve managerial capacity is a question for future research.

Our analysis has several limitations. The sample size is small, the data are cross-sectional, and personality traits were measured through self-reports rather than via staff observations. These characteristics limit our ability to draw causal conclusions, and the findings should be interpreted as correlations rather than causal effects. Our findings also come from a unique set of organizations operating in specific contexts, potentially limiting generalizability. In the LINKAGES program, DICs were overseen and supported by IPs, which in turn received oversight and assistance from country offices and headquarters. However, the use of average marginal effects and country fixed effects ensured that our personality estimates remained statistically stable and were identified based on within-country variation. Future studies using larger samples and longitudinal data could better characterize the relationships between personality traits and managerial capacity and examine how organizational structure and above-facility support interact with manager characteristics. Qualitative studies, such as Akeju et al. (2021) [60], could complement quantitative approaches.

## Conclusions

This study provides evidence of associations between manager personality traits and management levels in community-based facilities providing HIV services to key populations in Kenya and Malawi. Our findings suggest the potential value of considering personality traits in manager selection and management development and support. Studies with larger samples and longitudinal data are needed to better understand these relationships.

## Supporting information

S1 Table List of management practices for each management domain. Notes: All items coded as 1=Yes, 0=No
(DOCX)

S2 Table Coding and construction of the personality and meta-personality traits. Note: *Reverse items inverted the score: 1 = strongly agree & 7 = strongly disagree. Z-scores were calculated for all scores following Anderson’s (2008) [52] procedure.
(DOCX)

S3 Table Variables used for defining DIC clusters. (DOCX)
