# Supplementary material for: Personality traits and the managerial capacity of community-based facilities providing HIV services to key populations in Kenya and Malawi
Source: PLoS One. 2026 Jun 26;21(6):e0352752. doi: 10.1371/journal.pone.0352752 (PMC13308862; doi:10.1371/journal.pone.0352752)
Supplement: S1 Table — Notes: All items coded as 1=Yes, 0=No (DOCX) [file pone.0352752.s001.docx]

| **Management dimensions** | **Items** | **Number of items included in the score** |
| --- | --- | --- |
| **Target setting** | DIC-specific goals were set every year, for example number of individuals reached or testing rates | 7 |
|  | The DIC had goals related to the spending plan (budget). |  |
|  | The DIC set goals related to community involvement. |  |
|  | The DIC set goals related to marketing and demand generation. |  |
|  | The DIC set goals for individual staff members. |  |
|  | The DIC set goals at the team level. |  |
|  | A timeline was made and updated to reach the staff goals and targets. |  |
| **Performance monitoring** | **Activities carried out at the DIC during 2018- 2019** | 18 |
|  | The DIC was required to inform external entities about facility performance. |  |
|  | The DIC was required to inform external entities about staff performance. |  |
|  | The DIC informed external entities about their performance on meeting supply targets |  |
|  | The DIC informed external entities about their performance on meeting budgets and expenditures |  |
|  | The DIC, procedures were sent/shown to an external entity. |  |
|  | The DIC organized internal meetings to report DIC performance. |  |
|  | The in-country office supervised the frequency and content of meetings held at the DIC |  |
|  | The DIC was required to send evidence of meetings and agreements related to service provision |  |
|  | **External entity evaluation** |  |
|  | External entity evaluates the DIC in terms of: Stock of supplies |  |
|  | External entity evaluates the DIC in terms of: Staff performance |  |
|  | External entity evaluates the DIC in terms of: Budget and expenditures |  |
|  | External entity evaluates the DIC in terms of: Quality of care |  |
|  | External entity evaluates the DIC in terms of: DIC layout |  |
|  | **Periodic internal review evaluation** |  |
|  | The DIC engage in periodic internal review evaluation of: Stock of supplies |  |
|  | The DIC engage in periodic internal review evaluation of: Staff performance |  |
|  | The DIC engage in periodic internal review evaluation of: Budget and expenditures |  |
|  | The DIC engage in periodic internal review evaluation of: Quality of care |  |
|  | The DIC engage in periodic internal review evaluation of: DIC layout |  |
| **People management** | **Structures or activities that were normally presented or carried out at the DIC during 2018/2019** | 20 |
|  | The DIC evaluates the performance of its operational staff. |  |
|  | Staff members receive incentives or rewards to recognize their performance |  |
|  | Staff members receive sanctions for poor performance. |  |
|  | Staff that is not engaged or is partially engaged, receives the full salary at the month end |  |
|  | **Types of incentives or rewards implemented among staff during 2018/2019** |  |
|  | Time off |  |
|  | Verbal recognition |  |
|  | Written recognition / certificates |  |
|  | Monetary bonuses |  |
|  | Subsidized trainings/courses |  |
|  | Preferred schedule |  |
|  | Commodities (e.g., food basket) |  |
|  | **Types of incentives sanctions or rewards were implemented among staff during 2018/2019** |  |
|  | Verbal warning |  |
|  | Written warning |  |
|  | Forced leave or relocation |  |
|  | Pay reduction |  |
|  | Become ineligible for monetary bonuses (e.g., per diem, punctuality bonus) |  |
|  | Less desirable schedule |  |
|  | **Training** |  |
|  | DIC manager or any of the employees of the facility attended any training |  |
|  | The facility has training plans for clinical staff |  |
|  | During 2018-2019, the DIC manager was trained in topics related to general management |  |
| **Operations management** | **Structures or activities were normally present / carried out at the DIC during 2018-2019** | 11 |
|  | The DIC has an established schedule to perform organization-related activities, such as sorting, labelling, and filing documents (e.g., patient records, MoH registers) |  |
|  | The DIC has a dedicated space to store lab inputs (e.g., Rapid Diagnostic Tests (RDTs), lab reagents) |  |
|  | The DIC has a dedicated space to store drugs |  |
|  | Medical supplies are stored and organized, as soon as they arrive |  |
|  | There is a documented process that describes how to manage drug stock, RDTs and other lab inputs |  |
|  | The DIC does not feel crowded to walk by |  |
|  | The DIC usually operates in special schedules, to reach key populations (e.g., night shifts, weekends) |  |
|  | When the workload is heavy, the DIC employs locum nurses from other health facilities (e.g., government hospitals) |  |
|  | Procedures for staff are documented in a manual and easily accessible for consultation (SOPs) |  |
|  | Procedures are posted on the walls or blackboards of the DIC |  |
|  | Reminders are posted in a special place of the DIC (e.g., color-coded biohazard disposal reminders) |  |
| **Financial management** | **Structures or activities were normally present / carried out at the DIC during 2018-2019** | 7 |
|  | The DIC handle financial resources directly, e.g., revenue for providing health services, direct donations for purchasing inputs/paying utilities |  |
|  | The DIC provides a LINKAGES financial report every month |  |
|  | The DIC does a financial audit for itself every year on total facilities revenues and expenditures |  |
|  | Someone outside of the DIC (externals) come to do a financial audit every year on total DIC revenues and expenditures |  |
|  | The DIC elaborates spending plans (budget) each year |  |
|  | All payments are done through the bank / mobile money |  |
|  | The DIC has a dedicated bank account to manage DIC expenses |  |
| **Community engagement** | During 2018-2019, the DIC had a DIC committee / governing board | 4 |
|  | During 2018-2019, members of the community involved in the DIC committee / governing board |  |
|  | During 2018-2019, members of the community participated in the budget decision making process |  |
|  | During 2018-2019, members of the community participated in expenditures decisions? |  |
