# Supplementary material for: Personality traits and the managerial capacity of community-based facilities providing HIV services to key populations in Kenya and Malawi
Source: PLoS One. 2026 Jun 26;21(6):e0352752. doi: 10.1371/journal.pone.0352752 (PMC13308862; doi:10.1371/journal.pone.0352752)
Supplement: S2 Table — Note: *Reverse items inverted the score: 1 = strongly agree & 7 = strongly disagree. Z-scores were calculated for all scores following Anderson’s (2008) [52] procedure. (DOCX) [file pone.0352752.s002.docx]

| **Personality traits** | **Description of the score** | **Number of items included in the score** | **Items** | **Coding** |
| --- | --- | --- | --- | --- |
| Openness | It is a personality trait characterized by originality, curiosity, and ingenuity. Referred to as Culture because of its emphasis on intellectualism, polish, independence of mind, sophistication, and reflection. | 3 | I see myself as someone who is original and that comes up with new ideas. | Likert scale that goes from 1 to 7  1 = strongly agree 2 = disagree 3 = somewhat disagree 4 = neither agree nor disagree 5 = somewhat agree 6 = agree 7 = strongly agree |
|  |  |  | I see myself as someone who values artistic and aesthetic experiences. |  |
|  |  |  | I see myself as someone who has an active imagination. |  |
| Conscientiousness | Personality trait characterized by orderliness, responsibility, and dependability. Referred also as Dependability. | 3 | I see myself as someone who does a thorough job. |  |
|  |  |  | I see myself as someone who tends to be lazy*. |  |
|  |  |  | I see myself as someone who does things efficiently. |  |
| Extraversion | Personality trait characterized by talkativeness, assertiveness, and energy. Referred also as Surgency. | 3 | I see myself as someone who is talkative. |  |
|  |  |  | I see myself as someone who is outgoing/sociable. |  |
|  |  |  | I see myself as someone who is reserved*. |  |
| Agreeableness | Personality trait characterized by good-naturedness, cooperativeness, and trust. It can also be seen as a combination of friendliness and compliance | 3 | I see myself as someone who is sometimes rude to others*. |  |
|  |  |  | I see myself as someone who has a forgiving nature. |  |
|  |  |  | I see myself as someone who is considerate and kind to almost everyone. |  |
| Emotional Stability | Personality trait characterized by being level-headed, even in the face of challenges and threats. Is the opposite of Neuroticism. | 3 | I see myself as someone who worries a lot*. |  |
|  |  |  | I see myself as someone who gets nervous easily*. |  |
|  |  |  | I see myself as someone who remains calm in tense situations. |  |
| Plasticity |  | 2 | Openness Extraversion |  |
| Stability |  | 3 | Conscientiousness Agreeableness Emotional stability |  |
