# Supplementary material for: Personality traits and the managerial capacity of community-based facilities providing HIV services to key populations in Kenya and Malawi
Source: PLoS One. 2026 Jun 26;21(6):e0352752. doi: 10.1371/journal.pone.0352752 (PMC13308862; doi:10.1371/journal.pone.0352752)
Supplement: S3 Table — (DOCX) [file pone.0352752.s003.docx]

| **No.** | **Description** |
| --- | --- |
| 1 | The DIC is often required to revise and resubmit information about stock levels and consumption to implementing partners/donors at specified time intervals. |
| 2 | Does this DIC handle financial resources directly? |
| 3 | The DIC set goals for individual staff members. |
| 4 | A timeline was made and updated to reach the staff goals and targets. |
| 5 | During 2018-2019, did members of the community participate in the budget decision making process? |
| 6 | During 2018-2019, did members of the community participate in expenditures decisions? |
| 7 | The DIC had the authority to make decisions related to how the budget spend (such as reallocations of budget) without asking for approval from the country office (FHI360) |
| 8 | The DIC could apply for additional budget to cover unexpected expenses. |
