# Supplementary material for: Personality traits and the managerial capacity of community-based facilities providing HIV services to key populations in Kenya and Malawi
Source: PLoS One. 2026 Jun 26;21(6):e0352752. doi: 10.1371/journal.pone.0352752 (PMC13308862; doi:10.1371/journal.pone.0352752)
Supplement: Attachment — Submitted filename: PONE-D-26-01918_rev.pdf [file pone.0352752.s004.pdf]

PLOS One

Personality Traits and the Managerial Performance of Community-Based Facilities Providing HIV Services to Key Populations in Kenya and Malawi

--Manuscript Draft--

Manuscript Number:	PONE-D-26-01918
Article Type:	Research Article
Full Title:	Personality Traits and the Managerial Performance of Community-Based Facilities Providing HIV Services to Key Populations in Kenya and Malawi
Short Title:	Manager Personality Traits and the Managerial Performance of Community-Based Facilities
Corresponding Author:	Andrea Salas-Ortiz, PhD<br>University of York Centre for Health Economics<br>York, United Kingdom UNITED KINGDOM OF GREAT BRITAIN AND NORTHERN IRELAND
Keywords:	Personality traits; Management practices; HIV services; Community-based facilities; HIV key populations
Abstract:	Community-based facilities delivering HIV services to key populations often face significant operational and financial challenges. Thus, the role of effective management may be particularly critical in these organizations. A growing body of studies highlights the relevance of effective management in healthcare settings. However, effective management also depends on facility managers' managerial capacity and personality traits. This latter role has received no attention so far. To investigate the role of personality traits on managerial capacity across community-based facilities that provide HIV health services, from our population of 45 units in Kenya and Malawi, we first identify sub-groups of facilities that share similar characteristics and processes using cluster analysis. We then characterize the groups in terms of the level of managerial capacity. We make use of non-linear logit regression models to predict the probability of belonging to each of the groups as a function of both managers' personality traits while controlling for managers' schooling level, accumulated experience, and facilities' location characteristics. We proxy personality traits via the Big Five Inventory. We found two prototypes of organizations with statistically different levels of managerial capacity. One shows higher financial and people management skills, more practices related to performance monitoring and target setting, operations management, and higher levels of involvement with the local community to make financial-related decisions. When modelling the probability of belonging to the higher-management group, we found that educational achievement does not play a statistically significant role in this likelihood. However, a manager's experience working with HIV key populations and a stable personality consistently play a positive and statistically significant role in the probability of belonging to the high-managerial capacity type of organization. Higher managerial capacity is a key catalyst for organizational performance in contexts of limited resources. It can be boosted and enhanced not only through technical and cognitive skills but also by fostering and improving relevant personality traits.
Order of Authors:	Andrea Salas-Ortiz, PhD
	Marjorie Opuni
	José Luis Figueroa
	Louis Masankha Banda
	Alice Olawo
	Spy Munthali
	Julius Korir
	Barbara Nyambura Thirikwa
	Agatha Bula

Powered by Editorial Manager® and ProduXion Manager® from Aries Systems Corporation

	Navindra Persaud
	Sergio A. Bautista-Arredondo
**Additional Information:**	
**Question**	**Response**
**Financial Disclosure**<br><br>Enter a financial disclosure statement that describes the sources of funding for the work included in this submission. Review the submission guidelines for detailed requirements. View published research articles from *PLOS ONE* for specific examples.<br><br>This statement is required for submission and **will appear in the published article** if the submission is accepted. Please make sure it is accurate.<br><br>**Funded studies**<br>Enter a statement with the following details:<br>• Initials of the authors who received each award<br>• Grant numbers awarded to each author<br>• The full name of each funder<br>• URL of each funder website<br>• Did the sponsors or funders play any role in the study design, data collection and analysis, decision to publish, or preparation of the manuscript?<br><br>Did you receive funding for this work?	Yes
Please add funding details.<br>as follow-up to "**Financial Disclosure**<br><br>Enter a financial disclosure statement that describes the sources of funding for the work included in this submission. Review the submission guidelines for detailed requirements. View published research articles from *PLOS ONE* for specific examples.<br><br>This statement is required for submission and **will appear in the published article** if the submission is accepted. Please make	This study was funded by the Bill and Melinda Gates Foundation (grant: OPP1175038). The funding agency had no role in the study design, collection/analysis of data, data interpretation, or manuscript writing.

sure it is accurate.<br><br>**Funded studies**<br>Enter a statement with the following details:<br>• Initials of the authors who received each award<br>• Grant numbers awarded to each author<br>• The full name of each funder<br>• URL of each funder website<br>• Did the sponsors or funders play any role in the study design, data collection and analysis, decision to publish, or preparation of the manuscript?<br><br>Did you receive funding for this work?"	
Please select the country of your main research funder (please select carefully as in some cases this is used in fee calculation).<br>as follow-up to "**Financial Disclosure**<br><br>Enter a financial disclosure statement that describes the sources of funding for the work included in this submission. Review the submission guidelines for detailed requirements. View published research articles from *PLOS ONE* for specific examples.<br><br>This statement is required for submission and **will appear in the published article** if the submission is accepted. Please make sure it is accurate.<br><br>**Funded studies**<br>Enter a statement with the following details:<br>• Initials of the authors who received each award<br>• Grant numbers awarded to each author<br>• The full name of each funder<br>• URL of each funder website<br>• Did the sponsors or funders play any role in the study design, data collection and analysis, decision to publish, or preparation of the manuscript?<br><br>Did you receive funding for this work?"	UNITED STATES - US

**Competing Interests**<br><br>Use the instructions below to enter a competing interest statement for this submission. On behalf of all authors, disclose any competing interests that could be perceived to bias this work—acknowledging all financial support and any other relevant financial or non-financial competing interests.<br><br>This statement is required for submission and **will appear in the published article** if the submission is accepted. Please make sure it is accurate and that any funding sources listed in your Funding Information later in the submission form are also declared in your Financial Disclosure statement.<br><br>View published research articles from *PLOS ONE* for specific examples.<br><br>**NO authors have competing interests**<br><br>Enter: *The authors have declared that no competing interests exist.*<br><br>**Authors with competing interests**<br><br>Enter competing interest details beginning with this statement:<br><br>*I have read the journal's policy and the authors of this manuscript have the following competing interests: [insert competing interests here]*<br><br>* typeset	The authors declare that they have no competing interests.
**Ethics Statement**<br><br>Enter an ethics statement for this submission. This statement is required if the study involved:<br><br>• Human participants<br>• Human specimens or tissue<br>• Vertebrate animals or cephalopods	The study received ethical approval from the ethical review board of the National Institute of Public Health of Mexico (Number: 1554), the Kenya Medical Research Institute and the National Commission for Science, Technology, and Innovation (Protocol No. 4258), and the National Commission on Research Ethics in the Social Sciences and Humanities of Malawi (Protocol No. P/07/21/590). All DIC managers who participated in the management survey completed an electronic informed consent form. Throughout the analysis, the authors did not have access to information that could identify individual participants.

Powered by Editorial Manager® and ProduXion Manager® from Aries Systems Corporation

• Vertebrate embryos or tissues<br>• Field research<br><br>Write "N/A" if the submission does not require an ethics statement.<br><br>General guidance is provided below. Consult the submission guidelines for detailed instructions. **Make sure that all information entered here is included in the Methods section of the manuscript.**	

Powered by Editorial Manager® and ProduXion Manager® from Aries Systems Corporation

**Format for specific study types**<br><br>**Human Subject Research (involving human participants and/or tissue)**<br>• Give the name of the institutional review board or ethics committee that approved the study<br>• Include the approval number and/or a statement indicating approval of this research<br>• Indicate the form of consent obtained (written/oral) or the reason that consent was not obtained (e.g. the data were analyzed anonymously)<br><br>**Animal Research (involving vertebrate animals, embryos or tissues)**<br>• Provide the name of the Institutional Animal Care and Use Committee (IACUC) or other relevant ethics board that reviewed the study protocol, and indicate whether they approved this research or granted a formal waiver of ethical approval<br>• Include an approval number if one was obtained<br>• If the study involved *non-human primates*, add *additional details* about animal welfare and steps taken to ameliorate suffering<br>• If anesthesia, euthanasia, or any kind of animal sacrifice is part of the study, include briefly which substances and/or methods were applied<br><br>**Field Research**<br><br>Include the following details if this study involves the collection of plant, animal, or other materials from a natural setting:<br>• Field permit number<br>• Name of the institution or relevant body that granted permission	
**Data Availability**<br><br>Authors are required to make all data underlying the findings described fully available, without restriction, and from the time of publication. PLOS allows rare exceptions to address legal and ethical concerns. See the PLOS Data Policy and FAQ for detailed information.	Yes - all data are fully available without restriction

A Data Availability Statement describing where the data can be found is required at submission. Your answers to this question constitute the Data Availability Statement and **will be published in the article**, if accepted.<br><br>**Important:** Stating 'data available on request from the author' is not sufficient. If your data are only available upon request, select 'No' for the first question and explain your exceptional situation in the text box.<br><br>Do the authors confirm that all data underlying the findings described in their manuscript are fully available without restriction?	
**Describe where the data may be found in full sentences. If you are copying our sample text, replace any instances of XXX with the appropriate details.**<br><br>• If the data are **held or will be held in a public repository**, include URLs, accession numbers or DOIs. If this information will only be available after acceptance, indicate this by ticking the box below. For example: *All XXX files are available from the XXX database (accession number(s) XXX, XXX.).*<br>• If the data are all contained **within the manuscript and/or Supporting Information files**, enter the following: *All relevant data are within the manuscript and its Supporting Information files.*<br>• If neither of these applies but you are able to provide **details of access elsewhere**, with or without limitations, please do so. For example:<br><br>*Data cannot be shared publicly because of [XXX]. Data are available from the XXX Institutional Data Access / Ethics Committee (contact via XXX) for researchers who meet the criteria for access to confidential data.*<br><br>*The data underlying the results presented in the study are available from (include the name of the third party*	The data underlying the results presented in the study are available from Harvard Dataverse (https://dataverse.harvard.edu/dataset.xhtml?persistentId=doi:10.7910/DVN/NUWFU9.

and contact information or URL).<br>• This text is appropriate if the data are owned by a third party and authors do not have permission to share the data.<br><br>* typeset	
Additional data availability information:	

Manuscript
Click here to access/download;Manuscript;Personality Traits_v9Ene26.docx

Personality Traits and the Managerial Performance of Community-Based Facilities Providing HIV Services to Key Populations in Kenya and Malawi

Andrea Salas-Ortiz[1*], Marjorie Opuni[2], José Luis Figueroa[3], Louis Masankha Banda[5], Alice Olawo[6], Spy Munthali[7], Julius Korir[8], Barbara Nyambura Thirikwa[9], Agatha Bula[10], Navindra Persaud[11], Sergio Bautista-Arredondo[3,4§]

[1] Centre for Health Economics, University of York, York, United Kingdom

[2] Independent Consultant, Geneva, Switzerland

[3] Center for Evaluation Research and Surveys (CIEE), National Institute of Public Health (INSP), Cuernavaca, Mexico

[4] Economic Analysis Unit, Ministry of Health, Mexico City, Mexico.

[5] FHI 360, Lilongwe, Malawi

[6] FHI 360, Nairobi, Kenya

[7] University of Malawi, Zomba, Malawi

[8] Kenyatta University, Nairobi, Kenya

[9] Independent Consultant, Nairobi, Kenya

[10] UNC Project, Malawi

[11] FHI 360, Washington DC

*Corresponding author

Email: andrea.salasortiz@york.ac.uk

Abstract

Community-based facilities delivering HIV services to key populations often face significant operational and financial challenges. Thus, the role of effective management may be particularly critical in these organizations. A growing body of studies highlights the relevance of effective management in healthcare settings. However, effective management also depends on facility managers' managerial capacity and personality traits. This latter role has received no attention so far. To investigate the role of personality traits on managerial capacity across community-based facilities that provide HIV health services, from our population of 45 units in Kenya and Malawi, we first identify sub-groups of facilities that share similar characteristics and processes using cluster analysis. We then characterize the groups in terms of the level of managerial capacity. We make use of non-linear logit regression models to predict the probability of belonging to each of the groups as a function of both managers' personality traits while controlling for managers' schooling level, accumulated experience, and facilities' location characteristics. We proxy personality traits via the Big Five Inventory. We found two prototypes of organizations with statistically different levels of managerial capacity. One shows higher financial and people management skills, more practices related to performance monitoring and target setting, operations management, and higher levels of involvement with the local community to make financial-related decisions. When modelling the probability of belonging to the higher-management group, we found that educational achievement does not play a statistically significant role in this likelihood. However, a manager's experience working with HIV key populations and a *stable* personality consistently play a positive and statistically significant role in the probability of belonging to the high-managerial capacity type of organization. Higher managerial capacity is a key catalyst for organizational performance in contexts of limited resources. It can be boosted and enhanced not only through technical and cognitive skills but also by fostering and improving relevant personality traits.

Introduction

HIV services for key populations—men who have sex with men, female sex workers, people who inject drugs, and transgender women—tend to be most effective when delivered through community-based approaches that enhance accessibility and acceptability [1,2]. However, the community-based facilities delivering these services often face significant operational challenges. They provide a complex range of services, including clinical care and non-clinical interventions such as empowerment activities, stigma reduction programs, education, and peer outreach [3–5]. These services are typically delivered at fixed sites and directly within communities [6,7]. Community-based facilities frequently operate under significant human resource constraints, relying on diverse service providers, temporary workers, and community volunteers who need management support, technical guidance, training, and oversight from higher-level institutions [8]. In addition, many facilities also face financing challenges, often depending on multiple funding sources, each with its own performance monitoring and reporting requirements [9,10].

Given the challenges faced by community-based facilities providing HIV services to key populations, the role of effective management may be particularly critical in these organizations. A growing body of research highlights the importance of effective management in healthcare settings [11–13]. Studies have shown that more effective management is associated with superior clinical outcomes [14,15], better quality of care [12,15–21], and higher levels of efficiency [16,20,22,23].

Effective management is rooted partly in the implementation of good management practices including target setting, performance monitoring, people management, operations management, financial management, and community engagement [11,24–28]. Our previous research in Kenya and Malawi documented considerable variation in the management practices of health facilities, with some exhibiting high levels of management competence and others low levels [29]. At the same time, however, effective management also depends on the management capacity and personality traits of facility managers. Research suggests that both management practices and managerial behavior independently influence organizational performance [30].

Understanding the personality traits of community-based facility managers is important, as these likely influence how effectively managers deal with organizational challenges. Personality traits—patterns of thinking, feeling, and behaving not captured by educational achievements or intelligence quotient (IQ) [31,32]—may affect a manager's ability to coordinate complex service delivery, manage diverse staff, adapt to evolving community needs, and handle multi-faceted funding streams. While research has explored how cognitive skills influence managerial performance in healthcare [33], little attention has been paid to non-cognitive skills, such as personality traits, despite evidence of their importance in a variety of social and economic outcomes [31,34–36].

This paper examines how manager personality traits influence managerial performance in community-based facilities providing HIV services to key populations as part of the Linkages Across the Continuum of HIV Services for Key Populations Affected by HIV (LINKAGES) program in Kenya and Malawi. Services were delivered by local organizations called implementing partners (IPs) in facilities called drop-in centers (DICs) and in communities. In this paper, we investigate the relationship between managerial performance and the Big Five personality traits—Openness/Intellect, Conscientiousness, Extraversion, Agreeableness, and Emotional Stability—as well as the higher-order traits (or meta-traits) of Plasticity and Stability. To follow our aim, we first identify groups of DICs with similar managerial characteristics and then model the probability of DICs belonging to these groups based on managers' personality traits, controlling for education and experience. By identifying the traits associated with managerial performance in these organizations, we aim to contribute to the literature on health management in low-resource settings and provide insights to inform recruitment, training, and support strategies for community-based facility managers, ultimately improving the efficiency and effectiveness of HIV services for key populations.

Methods

Study Setting

This study analyzes data from the LINKAGES program in Kenya and Malawi. LINKAGES was funded by the United States Agency for International Development (USAID) through the United States President's Emergency Plan for AIDS Relief (PEPFAR) and administered by FHI 360 from

2014 to 2021. The program, implemented in Africa, Asia, and the Americas, aimed to reduce HIV transmission among key populations and improve their enrollment and retention in care and treatment. LINKAGES delivered a comprehensive package of HIV services to key populations. This package included the following clinical interventions: HIV testing services, antiretroviral therapy, post-exposure prophylaxis, pre-exposure prophylaxis, sexually transmitted infection treatment, sexual and reproductive health services, and the management of sexual violence. Alongside these clinical services, LINKAGES also provided non-clinical services such as empowerment programs and community engagement initiatives. LINKAGES also included pre-service activities such as population mapping and size estimation, as well as management and monitoring efforts at higher levels. Local community-based and key population-led organizations delivered HIV services at DICs and in communities, overseen by LINKAGES country offices and headquarters in the United States. A comprehensive overview of the LINKAGES program can be found elsewhere [37,38].

Data Collection

The data-collection instrument for this study has been described in detail elsewhere [29]. The survey tool was developed in collaboration with LINKAGES headquarters and the LINKAGES country offices in Kenya and Malawi. Data on DIC characteristics, management practices, and manager personality traits were collected through an online survey administered to DIC managers. The survey was applied from 1 November 2021 to 31 January 2022 using the Survey Monkey platform. We contacted 44 DIC managers (29 from Kenya and 15 from Malawi) and

achieved a 100% response rate. Our final sample included 45 observations since two DICs in Kenya were overseen by the same manager.

Ethical Clearance

The study received ethical approval from the ethical review board of the National Institute of Public Health of Mexico (Number: 1554), the Kenya Medical Research Institute and the National Commission for Science, Technology, and Innovation (Protocol No. 4258), and the National Commission on Research Ethics in the Social Sciences and Humanities of Malawi (Protocol No. P/07/21/590). All DIC managers who participated in the management survey completed an electronic informed consent form. Throughout the analysis, the authors did not have access to information that could identify individual participants.

Management Practices

Management practices were measured using the World Management Survey (WMS) framework [24] which focuses on four domains: target setting (establishing appropriate targets, tracking relevant outcomes, and ensuring alignment between targets and outcomes); performance monitoring (collecting and analyzing data to understand organization performance and identify improvement opportunities); people management (activities related to hiring, retaining, and rewarding high performance, and addressing underperformance), and operations management (standardizing processes and continuously improving organizational operations) [24,25,28]. Furthermore, two additional management domains were included in

this low-resource context: financial management (budgeting and financial accounting of revenues and expenses), and community engagement (developing and managing relationships with community members through community outreach, involvement of community leaders, and activities to ensure customer trust and satisfaction) [11,26,27]. A list of the items included in each management domain is provided in Table A1.

Scores for each management dimension were calculated by aggregating the items in Table A1, following Anderson's procedure [39]. For this, we first normalized each item by subtracting the mean and dividing by the standard deviation to obtain z-scores. We then created weighted average scores for each domain using the inverse of the covariance matrix of the transformed items within each domain. This approach required all items to be positively oriented, consistently indicating a better outcome. We calculated seven management scores for each DIC: six for the individual dimensions and an aggregated management score consolidating overall management capacity into a single indicator.

Personality Traits

Manager personality traits were measured using the Big Five personality inventory—the most prominent measurement system for personality traits [40,41]. This inventory assesses five personality traits: Openness/Intellect (intelligent, original, curious, and ingenious); Conscientiousness (orderly, responsible, dependable, and hard-working); Extraversion (talkative, assertive, and sociable); Agreeableness (cooperative, unselfish, good-natured,

trustworthy, friendly); and Emotional Stability[1] (predictable and consistent emotional reactions, even when facing challenges) [32,42–44]. More recent studies have shown that these five traits consistently relate to each other in a way that indicates two meta-personality traits: Stability (comprising Conscientiousness, Agreeableness, and Emotional Stability) and Plasticity (comprising openness/Intellect and Extraversion) [45–47]. While Stability involves the disposition to cope with stress, Plasticity involves the disposition towards exploration, flexibility and adaptation to novel situations [48].

We constructed aggregate scores for each personality and meta-personality trait using the same approach employed to construct the management scores—standardized weighted average z-scores were calculated using Anderson's procedure [39]. Table A2 shows all the items included to create each personality trait.

DIC Management Clusters

We used the data-driven method of k-means clustering, an unsupervised machine-learning method, to identify distinct groups of DICs based on multiple management characteristics simultaneously. This method partitions observations into a pre-specified number of non-overlapping clusters, where observations within each cluster share similar characteristics, while observations in different clusters do not. We used the eight management-related variables,

[1]Emotional Stability represents the positive side of what was originally termed Neuroticism (anxiety, vulnerability, irritability).

which are not part of the management scores, as inputs for the cluster analysis. These are shown in Table A3

Since the number of clusters is chosen arbitrarily by the algorithm, we determined the optimal number of clusters following the approach proposed by Makles (2012). This method consists of identifying several *k*-means solutions with different numbers of clusters and comparing them using within the sum of squares (WSS) and proportional reduction of error coefficient [49]. The *k*-means algorithm assigns each observation to a *k* cluster to minimize within-cluster variation, summed over all clusters. Our analysis identified two optimal DIC clusters as evidenced by the noticeable kink in the WSS at *k*=2 (Figure 1). Furthermore, η^2 points to a reduction of the WSS by approximately 40% at *k*=2 and minimal additional reduction for *k*>2.

Figure 1. WSS, log (WSS), η^2, and PRE for all *k* cluster solutions

To characterize the clusters, we compared the groups based on the distribution of the overall management score, the six management dimensions that comprise this score, and selected DIC and manager characteristics. Table 1 shows the differences in management and contextual characteristics between the two groups. We found statistically significant mean differences in the general management score and most management dimensions, except for performance monitoring and target setting. The groups were statistically similar in terms of DIC location and manager age and sex. Based on these results, we characterized the groups according to their observed level of management.

Table 1. Management scores and selected characteristics by DIC management cluster

DIC and manager's characteristics	*Lower* management level					*Higher* management level					Mean diff. p-value
	N	Mean	Median	Min	Max	N	Mean	Median	Min	Max	
Management											
**General score**	29	62.55	55.87	10.93	90	16	78.65	72	10.37	96.67	0.000
**Community involvement**	29	44.83	50.00	0.00	75.00	16	87.50	100.00	25.00	100.00	0.000
**Financial management**	29	34.48	0.00	0.00	100.00	16	61.71	87.51	0.00	100.00	0.044
**Operations management**	29	93.10	100.00	72.73	100.00	16	97.73	100.00	90.91	100.00	0.082
**People management**	29	30.69	30.00	10.00	65.00	16	43.13	42.50	20.00	80.00	0.008
**Performance monitoring**	29	79.12	83.33	44.44	100.00	16	85.42	91.67	50.00	100.00	0.237
**Target setting**	29	93.10	100.00	57.14	100.00	16	96.43	100.00	71.43	100.00	0.282
DIC location: Country											
Malawi	11	37.9%	-	-	-	4	25.0%	-	-	-	0.294
Kenya	18	62.1%	-	-	-	12	75.0%	-	-	-	
IP management type											
DICs from IPs with a unique DIC	5	17.2%	-	-	-	8	50.0%	-	-	-	0.025
DICs from IPs with two or more DICs	24	82.8%	-	-	-	8	50.0%	-	-	-	
Manager age group											
Young adults	16	55.2%	-	-	-	6	37.5%	-	-	-	0.205
Middle-aged adults	13	44.8%	-	-	-	10	62.5%	-	-	-	
Manager sex											
Male	13	44.8%	-	-	-	6	37.5%	-	-	-	0.438
Female	16	55.2%	-	-	-	10	62.5%	-	-	-	

Note: Diff. means difference.

Manager Personality Traits and DIC Management Clusters

We modeled the probability of a DIC belonging to either the lower or higher management cluster as a function of the manager's personality traits using non-linear logit models with several different specifications. First, we examined the probability of being in the higher management cluster based on the manager's formal education and the accumulated

experience of the manager *i* working with key populations (*EE* vector). We then added personality traits to the model by including the vector *PT,* which includes the two meta-traits Stability and Plasticity of manager *i*. In an alternative specification, we also incorporated all five Big Five personality traits.

To examine the sensitivity of our results, in subsequent specifications, we conditioned the role of manager personality traits on different DIC and manager characteristics, such as DIC location, IP management type, and the manager's age and sex. In all models, we clustered standard errors at the DIC level.

I) $g_k = \alpha + \beta_c EE_i + \epsilon_i$

II) $g_k = \alpha + \beta_c EE_i + \beta_{nc} PT_i + \epsilon_i$

III) $g_k = \alpha + \beta_c EE_i + \beta_{nc} PT_i + \gamma X_i^1 + \epsilon_i$

IV) $g_k = \alpha + \beta_c EE_i + \beta_{nc} PT_i + \gamma X_i^2 + \epsilon_i$

V) $g_k = \alpha + \beta_c EE_i + \beta_{nc} PT_i + \gamma X_i^3 + \epsilon_i$

VI) $g_k = \alpha + \beta_c EE_i + \beta_{nc} PT_i + \gamma X_i^4 + \epsilon_i$

VII) $g_k = \alpha + \beta_c EE_i + \beta_{nc} PT_i + \gamma X_i^5 + \epsilon_i$

VIII) $g_k = \alpha + \beta_c EE_i + \beta_{nc} PT_i + \gamma X_i^6 + \epsilon_i$

where:

X^1= country

X^2= DICs from IPs with two or more DICs.

X^3= Manager’s age.

X^4= Manager’s sex.

X^5= country, DICs from IPs with two or more DICs, manager’s age, and manager’s sex.

X^6= country, DICs from IPs with two or more DICs, and manager’s sex.

Results

Managers from both clusters displayed similar distributions and average levels of schooling and work experience but differed in their personality traits, as shown in Table 2. Managers in the higher management cluster showed significantly higher levels of Conscientiousness, Extraversion, and Emotional Stability compared to those in the lower management cluster, with these differences being statistically significant at the 5% statistical confidence level. Average levels of Openness/Intellect and Agreeableness did not differ significantly between clusters. However, when examining the two personality meta-traits, managers in the higher management cluster had, on average, higher levels of Plasticity and Stability than managers in the lower management cluster.

Table 2. Description of DIC clusters by manager personality traits and other characteristics

**Variables included**	***Lower* management level**					***Higher* management level**					**Mean diff. p-value**
	**N**	**Mean**	**SD**	**Min**	**Max**	**N**	**Mean**	**SD**	**Min**	**Max**	
**Manager personality traits**[a]											
Big Five personality traits											
Openness/Intellect	29	-0.22	1.04	-2.76	1.32	16	-0.04	1.14	-2.69	1.32	0.6098
Conscientiousness	29	-0.22	0.94	-2.86	0.98	16	0.37	0.70	-0.94	0.98	0.0323
Extraversion	29	-0.34	0.97	-2.32	1.70	16	0.43	0.85	-1.11	1.70	0.0106
Agreeableness	29	-0.10	1.12	-3.10	1.20	16	0.36	0.88	-1.11	1.20	0.1631

Emotional stability	29	-0.26	1.16	-3.10	1.19	16	0.46	0.72	-1.20	1.19	0.0293
Personality meta-traits											
Plasticity	29	-0.19	0.52	-1.24	0.73	16	0.12	0.52	-0.79	0.95	0.0562
Stability	29	-0.15	0.69	-1.71	0.96	16	0.33	0.54	-0.56	0.96	0.0206
**Control variables**											
Level of manager schooling											
High school, nursery school, some college credit, technical training, or associate degree	14	48.3%	-	-	-	7	43.8%	-	-	-	0.509
Bachelor's degree or Postgraduate degree	15	51.7%	-	-	-	9	56.3%	-	-	-	
DIC manager's experience											
Number of years of experience working with HIV Key Populations	29	5.9	5	2	11	16	6.75	6	4	9	0.2586

Notes: [a]Expressed in z-scores. Diff. means difference.

Tables 3 and 4 show regression results from different model specifications. Table 3 presents findings from models using the two meta-traits, while Table 4 shows results using the Big Five personality traits. Both education and experience working with key populations were positively associated with management performance, though the coefficients for the level of schooling were smaller than those of work experience (Tables 3 and 4). This relationship was consistent across all eight regression specifications.

The regression results also indicated that both meta-personality traits— Stability and Plasticity—were positively associated with the probability of belonging to the higher management cluster, with Stability showing a stronger effect. A one standard deviation increase in Stability was associated with a 4.8-fold increase in the probability of belonging to the higher management cluster. The model regressions using the Big Five personality traits (Table 4) were consistent with those using the meta-personality traits (Table 3). These results highlighted that conscientiousness was the key personality trait driving these results.

Table 3. Results from the non-linear logit models using two meta-traits

	Model specifications	I	II	III	IV	V	VI	VII	VIII
	**Variables included**				***Dependent variable: higher management =1***				
	Plasticity		2.345	2.403	2.841	2.322	2.413	3.676	3.686
**Meta-personality**			(2.554)	(2.517)	(3.342)	(2.536)	(2.759)	(4.494)	(4.526)
**traits**	Stability		4.850**	4.961**	4.217*	4.757**	4.544*	4.183	4.766*
			(3.623)	(3.847)	(3.575)	(3.717)	(3.806)	(4.154)	(4.081)
	Highest degree or level of schooling you have completed: bachelor's degree or Postgraduate degree	1.050	1.267	1.245	1.625	1.305	1.336	1.679	1.487
		(0.586)	(0.804)	(0.745)	(1.117)	(1.032)	(0.945)	(1.450)	(1.033)
	Manager years of experience [a]	2.065**	3.884***	3.979***	3.519***	3.736***	3.928***	4.163***	4.023***
		(0.747)	(1.510)	(1.369)	(1.136)	(1.467)	(1.461)	(1.365)	(1.338)
	Kenya (Ref: Malawi)			0.851				0.308	0.307
**Controls**				(0.822)				(0.366)	(0.325)
	DICs from IPs with two or more DICs (Ref: DICs from IPs with a unique DIC)				0.233*			0.124**	0.134**
					(0.198)			(0.122)	(0.132)
	Middle-aged adults (Ref: Young adults)					1.153		0.978	
						(0.973)		(0.992)	
	Female (Ref: Male)						1.282	1.703	
							(1.013)	(1.332)	
	Observations	45	45	45	45	45	45	45	45
	Pseudo R-squared	0.0521	0.238	0.238	0.292	0.238	0.239	0.321	0.315

Notes: Coefficients expressed in odds ratios. Standard errors in parentheses. *** p<0.01, ** p<0.05, * p<0.1. [a]Expressed in z-scores

Table 4. Results from the non-linear logit models using the Big Five inventory scores

	Model specifications	I	II	III	IV	V	VI	VII	VIII
	**Variables included**				***Dependent variable: higher management =1***				
**Personality traits**	Openness/Intellect		0.929	0.921	1.045	0.924	0.936	1.059	1.070
			(0.319)	(0.308)	(0.431)	(0.301)	(0.329)	(0.398)	(0.502)
	Conscientiousness		3.147**	3.306**	2.489*	3.089**	3.010*	2.580	2.775
			(1.691)	(1.900)	(1.311)	(1.777)	(1.695)	(1.870)	(1.809)
	Extraversion		2.053	2.198	2.007	2.056	2.115	2.597	2.583
			(1.254)	(1.250)	(1.321)	(1.259)	(1.342)	(1.703)	(1.596)
	Agreeableness		1.116	1.146	1.072	1.113	1.067	1.128	1.214
			(0.255)	(0.274)	(0.278)	(0.259)	(0.318)	(0.377)	(0.373)
	Emotional stability		1.356	1.342	1.749	1.359	1.350	1.787	1.844
			(0.373)	(0.378)	(0.687)	(0.368)	(0.364)	(0.732)	(0.763)
**Controls**	Highest degree or level of schooling you have completed: bachelor's degree or Postgraduate degree	1.050	1.434	1.394	1.895	1.475	1.585	2.059	1.649
		(0.586)	(0.917)	(0.872)	(1.325)	(1.118)	(1.123)	(1.906)	(1.102)
	Manager years of experience [a]	2.065**	3.411***	3.690***	2.739***	3.278***	3.456***	3.313***	3.290***
		(0.747)	(1.374)	(1.543)	(0.862)	(1.339)	(1.379)	(1.042)	(1.158)
	Kenya (Ref: Malawi)			0.665				0.204	0.204
				(0.656)				(0.230)	(0.212)
	DICs from IPs with two or more DICs (Ref: DICs from IPs with a unique DIC)				0.179			0.080***	0.085***
					(0.188)			(0.078)	(0.080)
	Middle-aged adults (Ref: Young adults)					1.144		1.020	
						(1.008)		(1.011)	
	Female (Ref: Male)						1.506	1.930	
							(1.448)	(2.042)	
	Observations	45	45	45	45	45	45	45	45
	Pseudo R-squared	0.0521	0.290	0.294	0.349	0.291	0.294	0.390	0.383

Notes: Coefficients expressed in odds ratios. Standard errors in parentheses. *** $p<0.01$, ** $p<0.05$, * $p<0.1$. [a]Expressed in z-scores

Discussion

This study examines the relationship between manager personality traits and management performance in 45 community-based facilities providing HIV services to key populations in Kenya and Malawi; we identified two distinct clusters of facilities with lower and higher management levels. We also found certain manager personality traits to be correlated with management level, suggesting that these traits may be important factors in determining the management performance of these facilities.

Overall, our results are consistent with previous research suggesting the need to extend human resource training beyond enhancing knowledge and skills to build non-cognitive and socio-emotional skills, to enhance attitudes and shift norms [50]. A key study finding was the strong association between manager Stability scores and management level, with facilities managed by managers with higher Stability scores almost five times more likely to be in the higher management cluster.

Our findings also suggest that the Stability meta-trait plays a more critical role than Plasticity in predicting managerial performance in the facilities studied. We hypothesize that Stability, which is associated with maintaining consistency and structure [45], may be particularly relevant in facilities managing the delivery of complex service to clients who are often marginalized, diverse staff with varying qualifications, and limited resources from diverse funding sources. In contrast, Plasticity—which reflects openness to new information [45] and

creativity [48]—may be less central to management performance in the LINKAGES context[2], where significant levels of above-facility oversight were provided by LINKAGES country offices and headquarters, likely streamlining new information for DIC managers.

Among the Big Five personality traits, while Openness/Intellect, Conscientiousness Extraversion, Agreeableness, and Emotional Stability were all positively associated with management level, only the association with Conscientiousness was statistically significant. This finding is consistent with previous research showing that Conscientiousness is a predictor of various economic outcomes [32,51–53]. Conscientious individuals, characterized by their organization, responsibility, and achievement orientation may be more likely to implement essential management practices, such as systematic monitoring, consistent staff supervision, and sound financial controls.

Our findings suggest several potential implications for the human resource management of community-based facilities providing HIV services to key populations. The associations we found between personality traits and management levels indicate that organizations might consider incorporating some assessment of personality traits when selecting potential managers [54]. Our findings also suggest it could be valuable to consider these characteristics in leadership development training and the design of support programs for these managers. Contrary to the belief that personality traits are stable over short periods, research shows that

[2] For example, delivery of health services by LINKAGES takes a project approach than would be the case in a standard or routine government facility, where managers are encouraged to be innovative/creative to cover for shortages in funding for activities.

personality traits are malleable and can be developed throughout life [55,56]. This suggests the potential for interventions aimed at developing personality traits to improve managerial capacity, though these interventions should be tailored to individuals' age and personality to be most effective [32].

Several limitations should be considered when interpreting these findings. The sample size is small, the cross-sectional nature of our data, and the fact that personality traits data were not collected via staff observations, but instead relied on self-reporting limits our ability to draw causal conclusions—the findings should be interpreted as correlations rather than causal effects Our findings come from a unique set of organizations operating in specific contexts, potentially limiting generalizability—the unique structure of the LINKAGES program where DICs were overseen and supported by IPs, which in turn received oversight and assistance from country offices and headquarters of the Ministry of Health. Future studies should be conducted using larger samples and longitudinal data to better understand the causal relationships between personality traits and management performance and examine how organizational structure and above-facility support interact with manager characteristics. Moreover, qualitative studies, e.g. Akeju et al. (2021), could complement quantitative approaches to provide richer insights into manager personality traits and management levels.

Conclusions

This study provides evidence of associations between manager personality traits and management levels in community-based facilities providing HIV services to key populations in

Kenya and Malawi. Our findings suggest the potential value of considering personality traits in manager selection and management development and support. Future studies using larger samples and longitudinal data are urgently needed in this area. Good management practices are a promising way to provide higher quality and lower cost health services in an increasingly resource-constrained environment.

Acknowledgments

We are grateful for the support and helpful comments on this analysis received from colleagues from FIH360, and managers and staff of the LINKAGES program in Kenya, and Malawi. We thank Alejandra Rodríguez-Atristain, Jorge Eduardo Sánchez-Morales and David Contreras-Loya for their assistance with data curation and analysis. We also thank the United States Agency for International Development (USAID) for supporting the LINKAGES program (Cooperative Agreement AID-OAA-A-14-00045).

References

1. International AIDS Society I. A decision framework for differentiated antiretroviral therapy delivery for key populations. Amsterdam; 2018. Available: https://differentiatedservicedelivery.org/Portals/0/adam/Content/2a0WxWUHfUKtul1mKWdmGQ/File/Decision%20Framework%20Key%20Population%20Web3.pdf

2. World Health Organisation W. Consolidated guidelines on HIV prevention, diagnosis, treatment and care for key populations. 2016. Available: https://www.who.int/publications-detail-redirect/9789241511124

3. Beattie TSH, Bhattacharjee P, Suresh M, Isac S, Ramesh BM, Moses S. Personal, interpersonal and structural challenges to accessing HIV testing, treatment and care services among female sex workers, men who have sex with men and transgenders in Karnataka state, South India. J Epidemiol Community Health. 2012;66: ii42–ii48. doi:10.1136/jech-2011-200475

4. Laga M, Vuylsteke B. Evaluating AVAHAN's design, implementation and impact: lessons learned for the HIV Prevention Community. BMC Public Health. 2011;11: S16. doi:10.1186/1471-2458-11-S6-S16

5. Vassall A, Chandrashekar S, Pickles M, Beattie TS, Shetty G, Bhattacharjee P, et al. Community mobilisation and empowerment interventions as part of HIV prevention for female sex workers in Southern India: a cost-effectiveness analysis. PloS One. 2014;9: e110562. doi:10.1371/journal.pone.0110562

6. Herce ME, Miller WM, Bula A, Edwards JK, Sapalalo P, Lancaster KE, et al. Achieving the first 90 for key populations in sub-Saharan Africa through venue-based outreach: challenges and opportunities for HIV prevention based on PLACE study findings from Malawi and Angola. J Int AIDS Soc. 2018;21 Suppl 5: e25132. doi:10.1002/jia2.25132

7. Weir SS, Figueroa JP, Scott M, Byfield L, Cooper CJ, Hobbs MC. Reaching key populations through key venues: Insights from the Jamaica HIV Prevention Program. PLOS ONE. 2018;13: e0206962. doi:10.1371/journal.pone.0206962

8. UNAIDS. UNAIDS guidance for partnerships with civil society, including people living with HIV and key populations. 2012. Available: https://www.unaids.org/en/resources/documents/2012/20120124_JC2236_guidance_partnership_civilsociety

9. Benotsch EG, Stevenson LY, Sitzler CA, Kelly JA, Makhaye G, Mathey ED, et al. HIV Prevention in Africa: Programs and Populations Served by Non-Governmental Organizations. J Community Health. 2004;29: 319–336. doi:10.1023/B:JOHE.0000025329.10411.2a

10. DiCarlo MC, Dallabetta GA, Akolo C, Bautista-Arredondo S, Digolo HV, Fonner VA, et al. Adequate funding of comprehensive community-based programs for key populations needed now more than ever to reach and sustain HIV targets. J Int AIDS Soc. 2022;25: e25967. doi:10.1002/jia2.25967

11. Bradley EH, Taylor LA, Cuellar CJ. Management Matters: A Leverage Point for Health Systems Strengthening in Global Health. Int J Health Policy Manag. 2015;4: 411–415. doi:10.15171/ijhpm.2015.101

12. Contreras-Loya D, Gertler P, Kwan A. Managerial Practices and Altruism in Health Care Delivery. USA; 2021. Available: https://www.dropbox.com/s/uz5vtksrft67ud1/Contreras_David_JMP_111521.pdf?dl=0

13. Lega F, Prenestini A, Spurgeon P. Is Management Essential to Improving the Performance and Sustainability of Health Care Systems and Organizations? A Systematic Review and a Roadmap for Future Studies. Value Health. 2013;16: S46–S51. doi:10.1016/j.jval.2012.10.004

14. Bloom N, Eifert B, Mahajan A, McKenzie D, Roberts J. Does Management Matter? Evidence from India. Q J Econ. 2013;128: 1–51. doi:10.1093/qje/qjs044

15. Dorgan S, Layton D, Bloom N, Homkes R, Sadun R. Management in healthcare : why good practice really matters. London : Centre for Economic Performance, LSE; 2010.

16. Chivardi C, Sosa AZ, Galárraga O, Sosa-Rubí SG. Efficiency, quality, and management practices in multidisciplinary and traditional diabetes healthcare services in Mexico. Health Serv Outcomes Res Methodol. 2023 [cited 25 Jul 2023]. doi:10.1007/s10742-023-00309-y

17. McConnell KJ, Lindrooth RC, Wholey DR, Maddox TM, Bloom N. Management Practices and the Quality of Care in Cardiac Units. JAMA Intern Med. 2013;173: 684–692. doi:10.1001/jamainternmed.2013.3577

18. McConnell KJ, Chang AM, Maddox TM, Wholey DR, Lindrooth RC. An exploration of management practices in hospitals. Healthcare. 2014;2: 121–129. doi:10.1016/j.hjdsi.2013.12.014

19. McConnell KJ, Lindrooth RC, Wholey DR, Maddox TM, Bloom N. Modern Management Practices and Hospital Admissions. Health Econ. 2016;25: 470–485. doi:10.1002/hec.3171

20. Sosa-Rubí SG, Bautista-Arredondo S, Chivardi-Moreno C, Contreras-Loya D, La Hera-Fuentes G, Opuni M. Efficiency, quality, and management practices in health facilities providing outpatient HIV services in Kenya, Nigeria, Rwanda, South Africa and Zambia. Health Care Manag Sci. 2021;24: 41–54. doi:10.1007/s10729-020-09541-1

21. Tsai TC, Jha AK, Gawande AA, Huckman RS, Bloom N, Sadun R. Hospital board and management practices are strongly related to hospital performance on clinical quality metrics. Health Aff Proj Hope. 2015;34: 1304–1311. doi:10.1377/hlthaff.2014.1282

22. Bautista-Arredondo S, Colchero MA, Amanze OO, Hera-Fuentes GL, Silverman-Retana O, Contreras-Loya D, et al. Explaining the heterogeneity in average costs per HIV/AIDS patient in Nigeria: The role of supply-side and service delivery characteristics. PLOS ONE. 2018;13: e0194305. doi:10.1371/journal.pone.0194305

23. Bautista-Arredondo S, La Hera-Fuentes G, Contreras-Loya D, Kwan A, Van Buren SJ, Amanze OO, et al. Efficiency of HIV services in Nigeria: Determinants of unit cost variation of HIV counseling and testing and prevention of mother-to-child transmission interventions. PloS One. 2018;13: e0201706. doi:10.1371/journal.pone.0201706

24. Bloom N, Lemos R, Sadun R, Scur D, Van Reenen J. JEEA-FBBVA Lecture 2013: The New Empirical Economics of Management. J Eur Econ Assoc. 2014;12: 835–876. doi:10.1111/jeea.12094

25. Bloom N, Sadun R, Van Reenen J. Does management really work? Harv Bus Rev. 2012;90: 77–80, 82, 148.

26. Mabuchi S, Alonge O, Tsugawa Y, Bennett S. Measuring management practices in primary health care facilities – development and validation of management practices scorecard in Nigeria. Glob Health Action. 2020;13: 1763078. doi:10.1080/16549716.2020.1763078

27. Macarayan EK, Ratcliffe HL, Otupiri E, Hirschhorn LR, Miller K, Lipsitz SR, et al. Facility management associated with improved primary health care outcomes in Ghana. PloS One. 2019;14: e0218662. doi:10.1371/journal.pone.0218662

28. Scur D, Sadun R, Van Reenen J, Lemos R, Bloom N. The World Management Survey at 18: lessons and the way forward. Oxf Rev Econ Policy. 2021;37: 231–258. doi:10.1093/oxrep/grab009

29. Salas-Ortiz A, Opuni M, Rodríguez-Atristain A, Figeroa JL, Sánchez-Morales JE, Bula AK, et al. Management practices in facilities providing HIV services to key populations in Kenya and Malawi: A descriptive analysis of management in community-based organizations. PLOS Glob Public Health. 2024;4: e0002813. doi:10.1371/journal.pgph.0002813

30. Bandiera O, Prat A, Hansen S, Sadun R. CEO Behavior and Firm Performance. J Polit Econ. 2020;128: 1325–1369. doi:10.1086/705331

31. Borghans L, Duckworth AL, Heckman JJ, Weel B ter. The Economics and Psychology of Personality Traits. J Hum Resour. 2008;43: 972–1059. doi:10.3368/jhr.43.4.972

32. Kautz T, Heckman JJ, Diris R, ter Weel B, Borghans L. Fostering and Measuring Skills: Improving Cognitive and Non-Cognitive Skills to Promote Lifetime Success. National Bureau of Economic Research; 2014. doi:10.3386/w20749

33. Janke K, Propper C, Sadun R. The Impact of CEOs in the Public Sector: Evidence from the English NHS. National Bureau of Economic Research; 2019. doi:10.3386/w25853

34. Deming DJ. The Growing Importance of Social Skills in the Labor Market. National Bureau of Economic Research; 2015. doi:10.3386/w21473

35. Heckman JJ, Rubinstein Y. The Importance of Noncognitive Skills: Lessons from the GED Testing Program. Am Econ Rev. 2001;91: 145–149.

36. Hilger A, Nordman CJ, Sarr LR. Cognitive and Non-Cognitive Skills, Hiring Channels, and Wages in Bangladesh. SSRN Electron J. 2018 [cited 17 Jul 2023]. doi:10.2139/ssrn.3205845

37. Opuni M, Figueroa JL, Sanchez-Morales JE, Salas-Ortiz A, Ochoa-Sanchez LE, Morales-Vazquez M, et al. The Cost of Providing Comprehensive HIV Services to Key Populations: An Analysis of the LINKAGES Program in Kenya and Malawi. Glob Health Sci Pract. 2023;11. doi:10.9745/GHSP-D-22-00538

38. Opuni M, Sanchez-Morales JE, Figueroa JL, Salas-Ortiz A, Banda LM, Olawo A, et al. Estimating the cost of HIV services for key populations provided by the LINKAGES program in Kenya and Malawi. BMC Health Serv Res. 2023;23: 337. doi:10.1186/s12913-023-09279-w

39. Anderson M. Multiple Inference and Gender Differences in the Effects of Early Intervention: A Reevaluation of the Abecedarian, Perry Preschool, and Early Training Projects. J Am Stat Assoc. 2008;103: 1481–1495.

40. Cobb-Clark DA, Schurer S. The stability of big-five personality traits. Econ Lett. 2012;115: 11–15. doi:10.1016/j.econlet.2011.11.015

41. Lang FR, John D, Lüdtke O, Schupp J, Wagner GG. Short assessment of the Big Five: robust across survey methods except telephone interviewing. Behav Res Methods. 2011;43: 548–567. doi:10.3758/s13428-011-0066-z

42. Digman JM. Personality Structure: Emergence of the Five-Factor Model. Annu Rev Psychol. 1990;41: 417–440. doi:10.1146/annurev.ps.41.020190.002221

43. Gosling SD, Rentfrow PJ, Swann WB. A very brief measure of the Big-Five personality domains. J Res Personal. 2003;37: 504–528. doi:10.1016/S0092-6566(03)00046-1

44. John OP, Naumann LP, Soto CJ. Paradigm shift to the integrative Big Five trait taxonomy: History, measurement, and conceptual issues. Handbook of personality: Theory and research, 3rd ed. New York, NY, US: The Guilford Press; 2008. pp. 114–158.

45. DeYoung CG. Higher-order factors of the Big Five in a multi-informant sample. J Pers Soc Psychol. 2006;91: 1138–1151. doi:10.1037/0022-3514.91.6.1138

46. DeYoung CG, Peterson JB, Higgins DM. Sources of Openness/Intellect: Cognitive and Neuropsychological Correlates of the Fifth Factor of Personality. J Pers. 2005;73: 825–858. doi:10.1111/j.1467-6494.2005.00330.x

47. Hirsh JB, Deyoung CG, Peterson JB. Metatraits of the Big Five differentially predict engagement and restraint of behavior. J Pers. 2009;77: 1085–1102. doi:10.1111/j.1467-6494.2009.00575.x

48. Feist GJ. Creativity and the Big Two model of personality: plasticity and stability. Curr Opin Behav Sci. 2019;27: 31–35. doi:10.1016/j.cobeha.2018.07.005

49. Makles A. Stata Tip 110: How to Get the Optimal K-Means Cluster Solution. Stata J. 2012;12. doi:10.1177/1536867X1201200213

50. Serneels P, Lievens T. Microeconomic institutions and personnel economics for health care delivery: a formal exploration of what matters to health workers in Rwanda. Hum Resour Health. 2018;16: 7. doi:10.1186/s12960-017-0261-9

51. Almlund M, Duckworth A, Heckman J, Kautz T. Personality Psychology and Economics. SSRN Electron J. 2011. doi:10.2139/ssrn.1765666

52. Dumfart B, Neubauer AC. Conscientiousness is the most powerful noncognitive predictor of school achievement in adolescents. J Individ Differ. 2016;37: 8–15. doi:10.1027/1614-0001/a000182

53. O’Connell M, Marks GN. Cognitive ability and conscientiousness are more important than SES for educational attainment: An analysis of the UK Millennium Cohort Study. Personal Individ Differ. 2022;188: 111471. doi:10.1016/j.paid.2021.111471

54. Schröder VS, Heimann AL, Ingold PV, Kleinmann M. Enhancing Personality Assessment in the Selection Context: A Study Protocol on Alternative Measures and an Extended Bandwidth of Criteria. Front Psychol. 2021;12. doi:10.3389/fpsyg.2021.643690

55. Roberts BW, Walton KE, Viechtbauer W. Patterns of mean-level change in personality traits across the life course: A meta-analysis of longitudinal studies. Psychol Bull. 2006;132: 1–25. doi:10.1037/0033-2909.132.1.1

56. Bleidorn W, Hopwood CJ, Back MD, Denissen JJA, Hennecke M, Hill PL, et al. Personality Trait Stability and Change. Personal Sci. 2021;2: e6009. doi:10.5964/ps.6009

57. Akeju D, Nance N, Salas-Ortiz A, Fakunmoju A, Ezirim I, Oluwayinka AG, et al. Management practices in community-based HIV prevention organizations in Nigeria. BMC Health Serv Res. 2021;21: 489. doi:10.1186/s12913-021-06494-1

Supporting information

S1 Table. List of management practices for each management domain

Notes: All items coded as 1=Yes, 0=No

S2 Table. Coding and construction of the personality and meta-personality traits

Note: *Reverse items inverted the score: 1=strongly agree & 7=strongly disagree. Z-scores were calculated for all scores following Anderson's (2008) procedure.

S3 Table. Variables used for defining DIC clusters

Click here to access/download;Figure;Figure 1.tiff

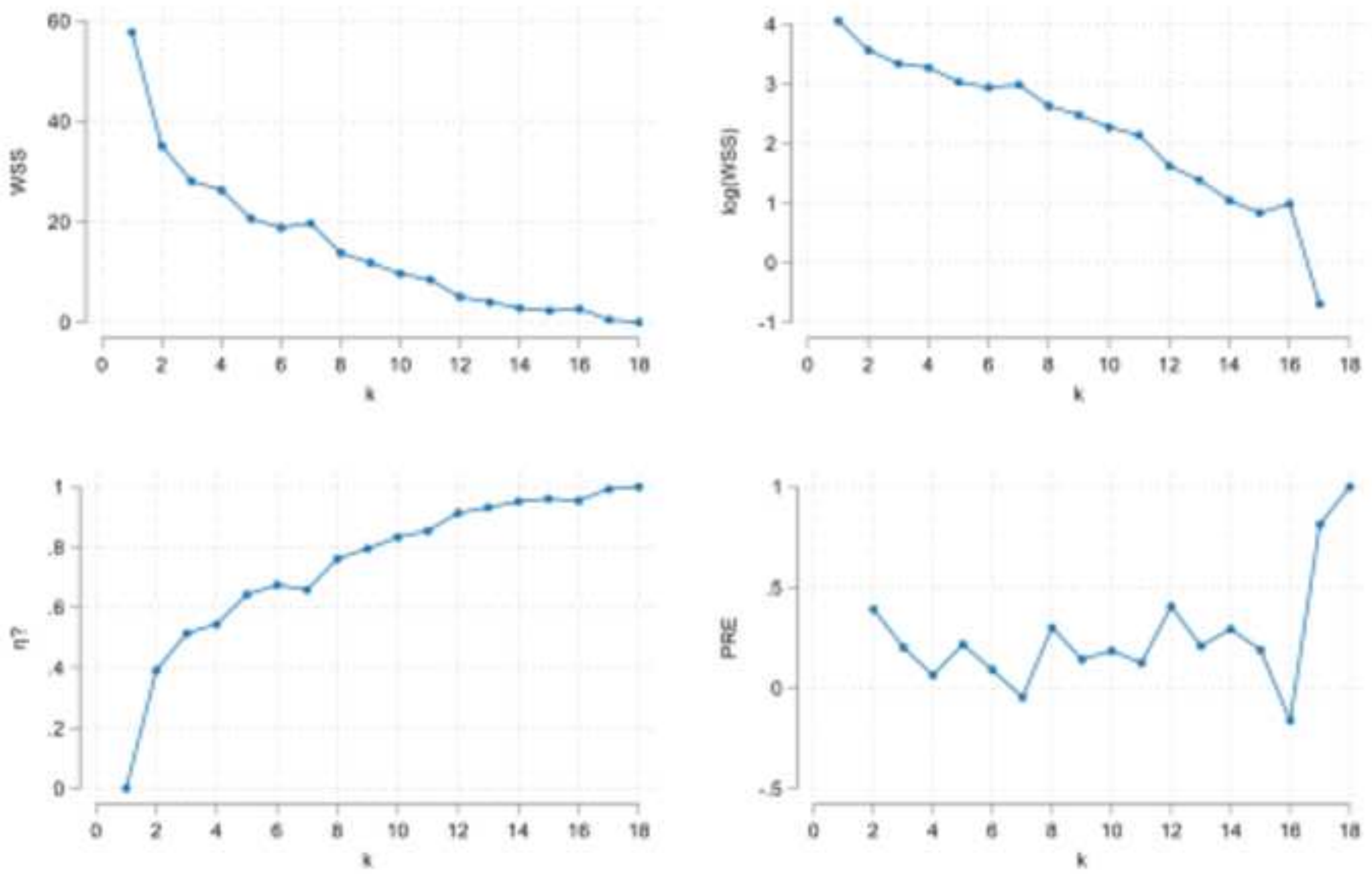

Click here to access/download
Supporting Information
S3_Table.docx

Click here to access/download
Supporting Information
S2_Table.docx

Click here to access/download
Supporting Information
S1_Table.docx
