# Supplementary material for: Personality traits and the managerial capacity of community-based facilities providing HIV services to key populations in Kenya and Malawi
Source: PLoS One. 2026 Jun 26;21(6):e0352752. doi: 10.1371/journal.pone.0352752 (PMC13308862; doi:10.1371/journal.pone.0352752)
Supplement: Attachment — Submitted filename: Reviewer Comments Updated.docx [file pone.0352752.s005.docx]

**Reviewer Comments on the Manuscript**

**Overall Assessment**

The manuscript explores an important and under-studied topic: the relationship between manager personality traits and managerial capacity in community-based HIV service facilities. The study contributes to the literature on health systems management in resource-limited settings. However, several conceptual, methodological, analytical, and reporting issues limit the strength of the conclusions.

**Study Title**

The title suggests managerial performance; however, the analysis appears to assess managerial capacity or adherence to management practices rather than performance. As such, the term ‘managerial performance’ may be misleading and should be reconsidered

**Recommendation**

- Consider revising the title to reflect the actual variable measured

**Abstract**

The abstract does not clearly specify the study design used

The statistical analysis sequence is not very clear. How was the transition from Cluster analysis to Logit regression made.

The abstract suggests: “personality traits can boost management capacity” but the study only shows associations, not causal relationships..

The statement: “stable personality consistently plays a positive and statistically significant role “is too general and not specific enough. Is there a statistical significance measure associated with this conclusion.

***Recommendation***

- The abstract should be strengthened by clearly defining the study design, clearly describing the key statistical analyses and the transition between them, and revising the conclusion to reflect association rather than causal relationships.

**Introduction**

While the introduction provides useful context on community-based HIV services, it requires stronger conceptual grounding. Specifically, it does not adequately explain based on existing literature or behavioral theories how personality traits may influence management performance in an HIV service delivery setting. The statement that personality traits in healthcare management have received ‘little attention so far’ (lines 90 – 93) should be further elaborated and supported.

Although the paper focuses on management capacity, its broader implications for HIV programme outcomes are not clearly articulated.

**Recommendation**

- The introduction if possible, should include a clearly defined conceptual model supported by relevant literature, a stronger theoretical explanation of how personality traits influence management performance, and a clearer articulation of the study’s relevance to health systems management, including how differences in management capacity relate to HIV programme outcomes.

**Methods**

**General comment**

The authors are encouraged to improve the clarity and organization of the methods section by structuring it under clear subheadings, including: Study Design, Study Setting, Sampling and Sample Size, measurement of variables, Data Collection Approaches, Data Analysis, and Ethical Considerations

***Study Setting***

The study setting had Limited explanation of Drop-In Centres

**Recommendation**

- The study setting section should provide a clearer and more detailed explanation of what Drop-In Centers are and their role in HIV service delivery for key populations, as the current description may not be sufficient for readers without prior experience in HIV programming or service delivery to fully understand their significance.

***Sampling and Sample Size***

Under the sampling and sample size section, the manuscript should provide stronger justification for the relatively small sample size, including how it was determined and whether it is sufficient to support the statistical analyses conducted. The authors should also discuss any constraints that limited sample expansion and the potential implications of the small sample size on the robustness and generalizability of the findings.

***Data Collection Approaches***

The study may be subject to self-reported survey bias, as managers assessed their own personality traits and management practices. This approach can introduce potential social desirability bias and self-evaluation bias, which may affect the accuracy and objectivity of the findings

**Recommendation**

- The authors should clarify what measures were implemented to minimize this bias

***Measurement of Management Practices***

While the World Management Survey framework was adapted, the aggregation procedure is not sufficiently described for readers unfamiliar with the method. In particular, the process used to derive and classify the management clusters lacks clarity and should be more explicitly explained.

**Recommendation**

- The authors should clearly describe the aggregation procedure and how management clusters were derived and classified to improve transparency and reproducibility.

***Personality Trait Measurement***

The manuscript does not specify which version of the Big Five Inventory was used (e.g., BFI-10, BFI-44), and it does not report the reliability of the personality scale. Also, the cultural validity of the instrument in the contexts of Kenya and Malawi has not been discussed.

**Recommendation**

- Specify the measurement tool by indicating which version of the Big Five Inventory was used, including appropriate references and justification for its selection.
- Address cultural validity by indicating the cultural appropriateness and validity of the instrument in the contexts of Kenya and Malawi, including any prior validation studies or adaptations made for these settings.

**Results**

The results section has several strengths, including clear presentation of cluster differences in tables and well-articulated comparisons of personality traits.

The authors should provide a clear explanation for running eight (8) models, in table 3 and 4 what goes into each model.

**Recommendations**

- The authors should provide a clear and detailed explanation of the modelling approach, including the rationale for running the eight models presented in Tables 3 and 4, and describe the variables and specifications included in each model

**Discussion**

**General comment**

The authors are encouraged to improve the structure of the manuscript by including clearly defined subsections following the discussion, specifically for Strengths, Limitations, and Recommendations, to enhance clarity and provide a balanced interpretation of the study findings.

**Limitations**

- The authors identified small sample size as a limitation, however, it would be important to clarify why the sample could not be expanded by including additional DICs, particularly given that the LINKAGES programme is implemented across multiple African countries. Providing justification for the sampling scope would help readers better understand the constraints and contextual factors influencing the study design.

**Major Revision**

The manuscript addresses an important topic and shows potential, but significant methodological, conceptual, and reporting gaps limit the clarity and robustness of the findings. Key issues include insufficient detail in the methods, unclear analytical procedures, concerns about small sample size and self-reported bias, and overinterpretation of associative results as causal. Substantial revisions are needed to improve transparency, rigor, and coherence before the manuscript can be considered for publication.
