# Supplementary material for: Personality traits and the managerial capacity of community-based facilities providing HIV services to key populations in Kenya and Malawi
Source: PLoS One. 2026 Jun 26;21(6):e0352752. doi: 10.1371/journal.pone.0352752 (PMC13308862; doi:10.1371/journal.pone.0352752)
Supplement: Attachment — Submitted filename: RR_letter_D26_01918_v27May26.docx [file pone.0352752.s007.docx]

PLOS ONE

PONE-D-26-01918

May 27^th^, 2026

Dear Professor Kwasi Torpey, MD, PhD, MPH

Academic Editor

***“Personality Traits and the Managerial Performance of Community-Based Facilities Providing HIV Services to Key Populations in Kenya and Malawi”***

We appreciate the time and effort that you and the reviewers spent reviewing our initial manuscript. The feedback has allowed us to refine our paper's content and presentation. We have revised our manuscript to address all the reviewers’ comments.

We have ensured that our manuscript meets PLOS ONE's style requirements and that the minimal data set necessary to replicate the study's findings is available in a public repository.

The submitted manuscript highlights all changes to the original text. We also include our point-by-point responses to the reviewers’ comments.

We thank you again for your time and consideration.

Yours sincerely,

Dr. Andrea Salas-Ortiz

Corresponding Author

**Responses to Editor and Reviewers**

We thank the Reviewers for their constructive comments and include below our responses in italics.

**Journal Requirements**

When submitting your revision, we need you to address these additional requirements.

1. Please ensure that your manuscript meets PLOS ONE's style requirements, including those for file naming. The PLOS ONE style templates can be found at

<https://journals.plos.org/plosone/s/file?id=wjVg/PLOSOne_formatting_sample_main_body.pdf> and

[https://journals.plos.org/plosone/s/file?id=ba62/PLOSOne_formatting_sample_title_authors_affiliations.pdf](https://track.editorialmanager.com/CL0/https:%2F%2Fjournals.plos.org%2Fplosone%2Fs%2Ffile%3Fid=ba62%2FPLOSOne_formatting_sample_title_authors_affiliations.pdf/1/010f019dbe12d7dd-b5f61263-3897-439d-b774-8b7d166443be-000000/cjYcHqrqv8_dt3twb0yDOB_6sU_OysgT1pm4r1riC3o=258)

*Response: Thanks. We have edited accordingly.*

2.  Thank you for uploading your study's underlying data set. Unfortunately, the repository you have noted in your Data Availability statement does not qualify as an acceptable data repository according to PLOS's standards.

At this time, please upload the minimal data set necessary to replicate your study's findings to a stable, public repository (such as figshare or Dryad) and provide us with the relevant URLs, DOIs, or accession numbers that may be used to access these data. For a list of recommended repositories and additional information on PLOS standards for data deposition, please see [https://journals.plos.org/plosone/s/recommended-repositories](https://track.editorialmanager.com/CL0/https:%2F%2Fjournals.plos.org%2Fplosone%2Fs%2Frecommended-repositories/1/010f019dbe12d7dd-b5f61263-3897-439d-b774-8b7d166443be-000000/PgN5kr1RZBrRLs6jGMkSSltjDUcvU_IxdX7BGMcFYKY=258).

*Response: Thanks. Harvard Dataverse is included in the list of recommended repositories. We uploaded the minimal dataset to replicate the findings. The URL is* [*https://doi.org/10.7910/DVN/MDVVVY*](https://doi.org/10.7910/DVN/MDVVVY)

3. If the reviewer comments include a recommendation to cite specific previously published works, please review and evaluate these publications to determine whether they are relevant and should be cited. There is no requirement to cite these works unless the editor has indicated otherwise.

*Response: Thanks. This was not the case*

**Reviewer:1**

1. This paper looks at a relatively under researched area for the context and would be a great addition to literature. The methods however needs revisions. There are a few missing pieces that the authors must consider to be able to reach the conclusions that they arrived at. These have been pointed out in the attached reviewed manuscript and partly include consideration of factors that may have influenced the observed relationships. Some of these have been published from the apparently the same dataset by the same authors. For instance, the authors had concluded in a recent publication that the level of above-site oversight might influence the management outcomes observed in the same facilities.

*Response: Thanks. Yes, previously it was found that organizational structure and oversight are relevant for management. In the light of that, in this paper we included the variable “IPs with 2+ DICs” as a control, which can be a proxy for oversight. This variable distinguishes between DICs that are part of a larger network (high potential for above-site oversight) versus those that are unique entities. By including this in the econometric models, we ensure that the reported effects of personality traits are independent of the influence of this oversight.*

*It is also worth noting that our results indicate that while oversight is important, managerial personality traits remain statistically significant predictors of a DIC being in the "high-management" cluster, even when these organizational factors are held constant. This suggests that personality traits add unique value to our understanding of facility performance beyond institutional design.*

2. It is also not clear why a clustering was done when individual managerial performance scores were available as well as individual personality trait scores.

*Response:* *We appreciate the opportunity to clarify why we used the cluster analysis method over a simpler way to dichotomize the dependent variable (for example, split the management score at some point of its distribution).*

*First, any split (p50, mean, p75, etc.) imposes a cut that may have no actual meaning, especially if the data is skewed. K-means is data-driven, minimizing within-cluster variance to find natural densities in the data. Using a clustering method, we identify functional profiles of management. This captures the complementarity between different management dimensions, which any distribution split would collapse into a single, linear, less meaningful binary variable.*

*Second, it is worth noting that the eight variables selected for clustering better capture managerial agency, whereas the broader management scores reflect procedural compliance. Clustering allows us to isolate the “agency/authority effect” within a DIC. This is conceptually more appropriate for our study, as personality traits (our independent variables) are more likely to manifest in managers who possess agency to make decisions, rather than those simply following standardized paperwork protocols.*

*Finally, this approach provides a robust internal validity check. The variables used for clustering were entirely separate from the variables used to construct the management scores. The fact that our clustering naturally resulted in groups with statistically significant differences in these management scores proves that we are predicting a coherent management profile rather than a hand-picked artefact of the distribution.*

3. There are other minor revisions that the authors are invited to consider in addition to the methodological concerns raised above. All these are in the attachment.

- Comments included in the PDF file

| **Page** | **Section** | **Location in Text** | **Reviewer Comment** | **Response** |
| --- | --- | --- | --- | --- |
| 10 | Abstract | Line 37 -- personality traits via the Big Five Inventory | Edit for clarity. | Edited to clarify |
| 10 | Abstract | Line 46 -- 'Higher managerial capacity is a key catalyst for organizational performance' | The manager belongs to a high managerial capacity organization? Not that the manager themselves have a high managerial capacity? | Edited to clarify |
| 10 | Abstract | Line 48 -- 'by fostering and improving relevant personality traits' | This does not come from your study. | Deleted |
| 11 | Introduction | Line 58 -- services sentence (community-based facilities face significant challenges) | Seems to be an abrupt sentence. Link to preceding sentence. | Edited to connect |
| 11 | Introduction | Line 61-62 -- 'Community-based facilities frequently operate under significant human resource constraints' | This also needs to be connected to preceding concepts. | Edited to connect |
| 11 | Introduction | Line 70 -- 'A growing body of research highlights the importance of effective management in organizations' | Effective management may be considered critical in all organizations. You may want to rephrase. | Edited to clarify |
| 12 | Introduction | Line 75 -- 'Effective management is rooted partly in the implementation of good management practices' | Rooted in or evidenced by? | Edited |
| 12 | Introduction | Line 81-82 -- 'Research suggests that both management capacity and personality traits of facility managers [matter]' | Any research suggesting this? | Edited/Added references |
| 13 | Introduction | Line 100 -- 'investigate the relationship between managerial performance and the Big Five personality traits' | Reference? | Added references |
| 13 | Introduction | Line 105-106 -- 'By identifying the traits associated with managerial performance' | Move to methods? | Edited |
| 13 | Introduction | Line 102 -- 'Stability -- as well as the higher-order traits (or meta-traits) of Plasticity and Stability' | Separate traits or aggregate traits? | Edited to clarify |
| 14 | Methods -- Data Collection | Line 136 -- SurveyMonkey platform, 44 DIC managers contacted | How were these selected? | Clarification added |
| 15 | Methods -- Data Collection | Line 138 -- 'two DICs in Kenya were overseen by the same manager' | Did you analyze the same manager twice? | Clarification added |
| 16 | Methods -- Management Domain Scores | Line 162 -- 'detailed information in each management domain is provided in Table A1' | S1? | Changed |
| 16 | Methods -- Personality Traits | Line 165 -- Anderson's (2008) procedure for normalizing items | You may want to include a brief justification. | Clarification added |
| 17 | Methods -- Cluster Analysis | Line 195-196 -- k-means clustering explanation | Why cluster? Since you had managerial performance for each facility and manager personality traits, was it possible to assess the managerial performance for each facility as per WMS and examine the influence of personality traits while controlling for confounders? | Clarification added |
| 19 | Methods -- Logit Models | Line 227-228 -- probability of being in the higher management cluster | Each DIC had a measure of managerial performance, a manager and the personality trait of that manager but this approach first clusters the facilities and then modeled a probability that the facility would fall in a cluster given the manager's personality trait. Does that seem like a roundabout way of examining what you had straightforward data to do? | Clarification added |
| 19 | Methods -- Logit Models | Line 228 -- manager's formal education and accumulated experience | Based on the context described, there are so many factors that could really influence any observed relationships. Did the managers get training on the job, how long were they on this job (not cumulative experience), did funding levels differ, what management support was available, was the intensity of support different from facility to facility? | Response below |
| 19 | Results -- Table | Table -- second set of observations (below the top half) | Include a heading for this set of observations that are different from the top half. | Included |
| 20 | Methods -- Logit Models | Line 229 -- manager experience with key populations | Were the managers at their jobs long enough to influence the managerial performance observed for their facilities? This is a critical missing piece. | Response below |
| 22 | Results -- Discussion | Line 282 -- Big Five results consistent with meta-trait results | Do the authors expect these to not be consistent? Are the meta-personality traits aggregates of the individual traits? | Addressed in the manuscript |
| 23 | Results -- Table | Page 23 -- table figure/number | 3 decimal spaces. | Done |
| 25 | Discussion | Line 315 -- 'funding sources' | Were these not LINKAGES-funded facilities? | Yes, edited accordingly. |
| 26 | Discussion | Line 318-319 -- 'likely streamlining new information for DIC managers' | This is an important factor to account for. Was it uniform across facilities regardless of performance? | We do not know and we have mentioned this in in text. |
| 26 | Discussion | Line 334 -- 'leadership development training and the design of support programs' | Did the authors review the training package for the managers? Was there training? Was it devoid of recommended considerations. | We do not have this information. However, here we are talking about community-based organizations generally, and we have clarified that. |
| 27 | Discussion | Line 338 -- 'interventions should be tailored to individuals' age and personality' | This was not explored at all in this study. | Deleted |
| 27 | Limitations | Line 345 -- 'effects Our findings come from...' | Full stop missing. | Added |
| 28 | Acknowledgements | Line 366 -- 'FIH360' | FHI 360. | Corrected |
| 28 | Acknowledgements | Line 369-370 -- USAID acknowledgement | You may want to direct this to the US Government who provided the funding through USAID. | Revised |

- Based on the context described - there are so many factors that could really influence any observed relationships and the authors will do well to include the data in their analysis. Did the managers get training on the job, how long were they on this job (not cumulative experience), did funding levels differ even though all were supported by LINKAGES, what management support was available, was the intensity of support different from facility to facility? The authors have published some findings from this data set and may be able to layer in some of those confounders here.

*Response:* *We appreciate the reviewer’s insight into the complex organizational factors that influence managerial capacity in these type of health organizations. While we acknowledge that variables such as job-specific training, tenure in the current role, and varying levels of IP support are theoretically relevant, we have intentionally limited the number of covariates in our econometric models to avoid overfitting and to preserve the statistical power of our analysis given the relatively small sample size. Adding further variables would significantly reduce our degrees of freedom and potentially lead to unreliable estimates. However, we believe these concerns are substantially mitigated by our existing model specifications. We included country fixed effects, capturing differences in national funding landscapes, Ministry of Health training protocols, and broader institutional support. This ensures that our results are identified through within-country variation, effectively controlling for systematic differences between the Kenyan and Malawian contexts. We also included IP-type, which proxy for the level of centralized administrative support and the standardized training regimes often associated with larger organizational structures. Finally, we have years of experience, which captures the accumulated human capital of the manager. By using these broader categorical controls, we maintain a parsimonious model that captures the latent influence of the factors mentioned by the reviewer without compromising the integrity of our statistical findings.*

- Were the managers at their jobs long enough to influence the managerial performance observed for their facilities? This is a critical missing piece.

*Response:* *We agree that the duration of a manager’s influence is an important consideration. While our data does not include a specific measure of tenure at the current facility, we believe this factor is effectively captured by our measure of years of experience. As shown in the regression results, this variable was a significant and robust predictor of belonging to the higher management cluster across all specifications. This suggests that the management practices we observed are a function of the manager’s accumulated expertise.*

**Reviewer:2**

The manuscript explores an important and under-studied topic: the relationship between manager personality traits and managerial capacity in community-based HIV service facilities. The study contributes to the literature on health systems management in resource-limited settings. However, several conceptual, methodological, analytical, and reporting issues limit the strength of the conclusions.

1. **Study Title**

The title suggests managerial performance; however, the analysis appears to assess managerial capacity or adherence to management practices rather than performance. As such, the term ‘managerial performance’ may be misleading and should be reconsidered. **Recommendation**. Consider revising the title to reflect the actual variable measured.

*Response: Thanks. The title of the manuscript is now: “Personality Traits and the Managerial Capacity of Community-Based Facilities Providing HIV Services to Key Populations in Kenya and Malawi”*

2. **Abstract**

The abstract does not clearly specify the study design used. The statistical analysis sequence is not very clear. How was the transition from Cluster analysis to Logit regression made. The abstract suggests: “personality traits can boost management capacity” but the study only shows associations, not causal relationships. The statement: “stable personality consistently plays a positive and statistically significant role “is too general and not specific enough. Is there a statistical significance measure associated with this conclusion. ***Recommendation***. The abstract should be strengthened by clearly defining the study design, clearly describing the key statistical analyses and the transition between them, and revising the conclusion to reflect association rather than causal relationships.

*Response: We thank the reviewer for these suggestions. We have revised the abstract.*

*The study design is now stated clearly as a cross-sectional, quantitative analysis of 45 facilities providing HIV services to key populations in Kenya and Malawi. The analysis is now described in two stages. We first use k-means cluster analysis on management practice data to identify subgroups of facilities with similar management profiles. We then use cluster membership as the dependent variable in logit regression models, estimating the probability that a facility belongs to the higher-managerial-capacity cluster as a function of managers' personality traits, controlling for education, experience, and facility characteristics. We have revised the language throughout the abstract to reflect associations rather than causal relationships.*

3. **Introduction**

While the introduction provides useful context on community-based HIV services, it requires stronger conceptual grounding. Specifically, it does not adequately explain based on existing literature or behavioral theories how personality traits may influence management performance in an HIV service delivery setting. The statement that personality traits in healthcare management have received ‘little attention so far’ (lines 90 – 93) should be further elaborated and supported. Although the paper focuses on management capacity, its broader implications for HIV programme outcomes are not clearly articulated. **Recommendation**. The introduction if possible, should include a clearly defined conceptual model supported by relevant literature, a stronger theoretical explanation of how personality traits influence management performance, and a clearer articulation of the study’s relevance to health systems management, including how differences in management capacity relate to HIV programme outcomes.

*Response: We thank the reviewer for these suggestions and we have revised the introduction to address them.*

*We have added Trait Activation Theory (Tett & Burnett, 2003) as the theoretical framework for the personality–management relationship. The theory holds that personality traits translate into work behaviors when the demands of the role activate them. We apply it to community-based HIV service delivery by describing the demands managers face: coordinating complex services, managing diverse staff, handling multiple funding streams, and responding to the needs of communities that face stigma and legal barriers to care (paragraph 4). We also reference Trait Activation Theory in the discussion when interpreting why Stability is more strongly associated with managerial capacity than Plasticity.*

*To better support the gap statement, we have revised paragraph 3. We now note that manager behavior has been shown to shape organizational performance in both the private sector and public health systems, and that personality traits have been examined more in relation to leadership effectiveness than to management capacity in health facilities.*

*On the link to HIV program outcomes, we are not aware of studies that directly link facility-level management capacity to outcomes. We note in paragraph 2 the evidence that more effective management is associated with higher efficiency in HIV services, and that a study of voluntary medical male circumcision found no statistically significant relationship between management practices and efficiency. This is the most we can say.*

4. **Methods**

**General comment.** The authors are encouraged to improve the clarity and organization of the methods section by structuring it under clear subheadings, including: Study Design, Study Setting, Sampling and Sample Size, measurement of variables, Data Collection Approaches, Data Analysis, and Ethical Considerations.

*Response: We agree that a more structured Methods section will improve the readability and transparency of the study. Thus, we have restructured the section to follow the suggested subheadings.*

5. ***Study Setting***

The study setting had Limited explanation of Drop-In Centres. **Recommendation**. The study setting section should provide a clearer and more detailed explanation of what Drop-In Centers are and their role in HIV service delivery for key populations, as the current description may not be sufficient for readers without prior experience in HIV programming or service delivery to fully understand their significance.

*Response: We agree that a clearer explanation of Drop-In Centers (DICs) is essential for readers who may not be familiar with specialized HIV programming for key populations. As suggested, we have expanded the Study Setting section.*

6. ***Sampling and Sample Size***

Under the sampling and sample size section, the manuscript should provide stronger justification for the relatively small sample size, including how it was determined and whether it is sufficient to support the statistical analyses conducted. The authors should also discuss any constraints that limited sample expansion and the potential implications of the small sample size on the robustness and generalizability of the findings.

*Response: The sample was determined by the scope of a broader costing project, which mapped all DICs that were part of the LINKAGES program. Thus, the sample size was dictated by the total number of operational facilities under that project rather than a discretionary choice by the authors. By surveying all 44 eligible managers and achieving a 100% response rate, our data constitutes an exhaustive look at this specific program. To ensure statistical robustness despite the number of observations, we utilized meta-trait aggregation to reduce dimensionality and preserve degrees of freedom. We know also show the Average Marginal Effects (AME) provide estimates less sensible to outliers. While the specialized nature of these facilities may limit direct generalizability to other public health settings, the findings provide a robust and novel analysis of the association between personality traits and managerial capacity.*

7. ***Data Collection Approaches***

The study may be subject to self-reported survey bias, as managers assessed their own personality traits and management practices. This approach can introduce potential social desirability bias and self-evaluation bias, which may affect the accuracy and objectivity of the findings. **Recommendation**. The authors should clarify what measures were implemented to minimize this bias

*Response: We acknowledge the potential for self-reported bias. However, we employed Anderson’s (2008) inverse-covariance weighting procedure to aggregate the personality scores. This method statistically mitigates bias by down-weighting variables that are highly redundant or strongly correlated—patterns often associated with consistent over-reporting—thereby reducing the influence of generalized noise on the final indices. Also note that our dependent variable is derived from an unsupervised machine-learning clustering algorithm (k-means) rather than a hand-picking metric. Because this classification is based on the multi-dimensional configuration of all inputs simultaneously, it is significantly robust to self-reporting: strategic manipulation of the resulting management clusters is highly improbable. These measures ensure that our results reflect the latent patterns in the data rather than systematic reporting bias.*

8. ***Measurement of Management Practices***

While the World Management Survey framework was adapted, the aggregation procedure is not sufficiently described for readers unfamiliar with the method. In particular, the process used to derive and classify the management clusters lacks clarity and should be more explicitly explained. **Recommendation**. The authors should clearly describe the aggregation procedure and how management clusters were derived and classified to improve transparency and reproducibility.

*Response: Thanks for the comment. We have significantly expanded the "Dependent Variable: Management Clusters" subsubsection within the Measurement of Variables subsection to provide a transparent explanation of our procedure. We explained that, first, we identified managerial capacity profiles via clustering analysis and using eight specific variables measuring managerial agency as inputs (Table S3). Second, we characterized and validated the clusters by comparing them against broader management scores (those based on the World Management Survey. Table S1). Crucially, these eight clustering inputs were excluded from the construction of the overall management scores to avoid circularity.*

9. ***Personality Trait Measurement***

The manuscript does not specify which version of the Big Five Inventory was used (e.g., BFI-10, BFI-44), and it does not report the reliability of the personality scale. Also, the cultural validity of the instrument in the contexts of Kenya and Malawi has not been discussed. **Recommendation**. Specify the measurement tool by indicating which version of the Big Five Inventory was used, including appropriate references and justification for its selection. Address cultural validity by indicating the cultural appropriateness and validity of the instrument in the contexts of Kenya and Malawi, including any prior validation studies or adaptations made for these settings.

*Response: We have updated the manuscript to specify that manager personality traits were measured using the BFI-15, a validated 15-item abbreviated version of the Big Five. Regarding the reliability of the scale, we acknowledge the reviewer's request for internal consistency measures. However, because each of the five traits is represented by only three items (Table S2), Cronbach’s alpha is known to be an unstable and often misleading indicator of reliability. Instead, we followed the established literature in personality psychology, which suggests that these five traits aggregate into two higher-order meta-traits. By analyzing these meta-traits, we provide a more robust statistical foundation for our regressions than the individual 3-item subscales would allow.*

*To address the concern regarding cultural validity and potential response bias in Kenya and Malawi, please note that we used Anderson’s (2008) inverse-covariance weighting to aggregate the traits. This procedure indirectly mitigates cultural response patterns by down-weighting redundant information and focusing on the unique variance of each trait. Also, all models include country fixed effects. This ensures that any country-level cultural traits or systematic tendencies to over- or under-report certain characteristics are absorbed by the country dummies, effectively mitigating concerns regarding scale comparability and cultural validity across the two settings.*

10. **Results**

The results section has several strengths, including clear presentation of cluster differences in tables and well-articulated comparisons of personality traits. The authors should provide a clear explanation for running eight (8) models, in table 3 and 4 what goes into each model. **Recommendations**. The authors should provide a clear and detailed explanation of the modelling approach, including the rationale for running the eight models presented in Tables 3 and 4, and describe the variables and specifications included in each model

*Response: We thank the reviewer for the opportunity to clarify our modeling approach. We have revised the methods section: Econometric Analysis subsection to provide a clear rationale for our modeling strategy. We adopted a stepwise sensitivity approach across eight specifications to rigorously test the stability of the relationship between manager personality and managerial capacity. Furthermore, we have clarified our use of dual reporting: we provide Odds Ratios to show relative likelihood and Average Marginal to provide a robust, population-averaged estimate of the actual probability shifts. This ensures that our findings are not only statistically significant but also practically interpretable.*

11. **Discussion**

**General comment.** The authors are encouraged to improve the structure of the manuscript by including clearly defined subsections following the discussion, specifically for Strengths, Limitations, and Recommendations, to enhance clarity and provide a balanced interpretation of the study findings. **Limitations.** The authors identified small sample size as a limitation, however, it would be important to clarify why the sample could not be expanded by including additional DICs, particularly given that the LINKAGES programme is implemented across multiple African countries. Providing justification for the sampling scope would help readers better understand the constraints and contextual factors influencing the study design.

*Response: We have restructured the discussion section. Regarding the sample expansion, it is important to clarify that this study was an analysis nested within a specific, multi-year LINKAGES costing study conducted in 2018/2019. The geographic scope of that study was restricted by its funding mandate, specifically to Kenya and Malawi. Given this, the sample could not be expanded to other countries where the costing study did not take place. However, within these two countries, we achieved a 100% response rate, covering the entire operational universe of LINKAGES-funded DICs. Therefore, the sample does not represent a subset of a larger available pool of sites, but a programmatic census of the initiative in these two nations. We have added this to the manuscript.*
