# Supplementary material for: Personality traits and the managerial capacity of community-based facilities providing HIV services to key populations in Kenya and Malawi
Source: PLoS One. 2026 Jun 26;21(6):e0352752. doi: 10.1371/journal.pone.0352752 (PMC13308862; doi:10.1371/journal.pone.0352752)
Supplement: Peer review history

# Peer review: Decision Letter 0

**DOI:** 10.1371/journal.pone.0352752.r001

**Authors:** Kwasi Torpey

24 Apr 2026

PONE-D-26-01918

Personality Traits and the Managerial Performance of Community-Based Facilities Providing HIV Services to Key Populations in Kenya and Malawi

PLOS One

Dear Dr. Salas-Ortiz,

Thank you for submitting your manuscript to PLOS ONE. After careful consideration, we feel that it has merit but does not fully meet PLOS ONE’s publication criteria as it currently stands. Therefore, we invite you to submit a revised version of the manuscript that addresses the points raised during the review process.

The authors are advised to provide additional methodological details and ensure there is clarity in the method sections. Furthermore, issues relating to managerial performance and managerial capacity needs to be clarified for the benefit of readers.

Please submit your revised manuscript by Jun 08 2026 11:59PM. If you will need more time than this to complete your revisions, please reply to this message or contact the journal office at plosone@plos.org. When you're ready to submit your revision, log on to https://www.editorialmanager.com/pone/ and select the 'Submissions Needing Revision' folder to locate your manuscript file.

Please include the following items when submitting your revised manuscript:

- A letter that responds to each point raised by the academic editor and reviewer(s). You should upload this letter as a separate file labeled 'Response to Reviewers'.
- A marked-up copy of your manuscript that highlights changes made to the original version. You should upload this as a separate file labeled 'Revised Manuscript with Track Changes'.
- An unmarked version of your revised paper without tracked changes. You should upload this as a separate file labeled 'Manuscript'.

If you would like to make changes to your financial disclosure, please include your updated statement in your cover letter. Guidelines for resubmitting your figure files are available below the reviewer comments at the end of this letter.

If applicable, we recommend that you deposit your laboratory protocols in protocols.io to enhance the reproducibility of your results. Protocols.io assigns your protocol its own identifier (DOI) so that it can be cited independently in the future. For instructions see: https://journals.plos.org/plosone/s/submission-guidelines#loc-laboratory-protocols. Additionally, PLOS ONE offers an option for publishing peer-reviewed Lab Protocol articles, which describe protocols hosted on protocols.io. Read more information on sharing protocols at https://plos.org/protocols?utm_medium=editorial-email&utm_source=authorletters&utm_campaign=protocols.

As the corresponding author, your ORCID iD is verified in the submission system and will appear in the published article. PLOS supports the use of ORCID, and we encourage all coauthors to register for an ORCID iD and use it as well. Please encourage your coauthors to verify their ORCID iD within the submission system before final acceptance, as unverified ORCID iDs will not appear in the published article. *Only*  the individual author can complete the verification step; PLOS staff *cannot*  verify ORCID iDs on behalf of authors.

We look forward to receiving your revised manuscript.

Kind regards,

Professor Kwasi Torpey, MD PhD MPH

Academic Editor

PLOS One

Journal Requirements:

When submitting your revision, we need you to address these additional requirements.

1. Please ensure that your manuscript meets PLOS ONE's style requirements, including those for file naming. The PLOS ONE style templates can be found at

https://journals.plos.org/plosone/s/file?id=wjVg/PLOSOne_formatting_sample_main_body.pdf and

https://journals.plos.org/plosone/s/file?id=ba62/PLOSOne_formatting_sample_title_authors_affiliations.pdf

2.  Thank you for uploading your study's underlying data set. Unfortunately, the repository you have noted in your Data Availability statement does not qualify as an acceptable data repository according to PLOS's standards.

At this time, please upload the minimal data set necessary to replicate your study's findings to a stable, public repository (such as figshare or Dryad) and provide us with the relevant URLs, DOIs, or accession numbers that may be used to access these data. For a list of recommended repositories and additional information on PLOS standards for data deposition, please see https://journals.plos.org/plosone/s/recommended-repositories.

If the reviewer comments include a recommendation to cite specific previously published works, please review and evaluate these publications to determine whether they are relevant and should be cited. There is no requirement to cite these works unless the editor has indicated otherwise.

Additional Editor Comments:

The manuscript titled " Personality Traits and Managerial Performance of Community Based Facilities Providing HIV Services to key Populations in Kenya and Malawi" provides good insights in a rather underresearched area. Though well written, there are areas that require improvement before being accepted. These include provide methodological details as well as providing more clarity. The authors are expected to address the issues pertaining to managerial performance vis a vis managerial capacity to ensure audience are clear in the outcomes.

[Note: HTML markup is below. Please do not edit.]

Reviewers' comments:

Reviewer's Responses to Questions

**Comments to the Author**

1. Is the manuscript technically sound, and do the data support the conclusions?

The manuscript must describe a technically sound piece of scientific research with data that supports the conclusions. Experiments must have been conducted rigorously, with appropriate controls, replication, and sample sizes. The conclusions must be drawn appropriately based on the data presented.

Reviewer #1: Partly

Reviewer #2: Yes

**********

2. Has the statistical analysis been performed appropriately and rigorously?

Reviewer #1: Yes

Reviewer #2: Yes

**********

3. Have the authors made all data underlying the findings in their manuscript fully available?

The PLOS Data policy requires authors to make all data underlying the findings described in their manuscript fully available without restriction, with rare exception (please refer to the Data Availability Statement in the manuscript PDF file). The data should be provided as part of the manuscript or its supporting information, or deposited to a public repository. For example, in addition to summary statistics, the data points behind means, medians and variance measures should be available. If there are restrictions on publicly sharing data—e.g. participant privacy or use of data from a third party—those must be specified.

Reviewer #1: No

Reviewer #2: No

**********

4. Is the manuscript presented in an intelligible fashion and written in standard English?

PLOS ONE does not copyedit accepted manuscripts, so the language in submitted articles must be clear, correct, and unambiguous. Any typographical or grammatical errors should be corrected at revision, so please note any specific errors here.

Reviewer #1: Yes

Reviewer #2: Yes

**********

5. Review Comments to the Author

Please use the space provided to explain your answers to the questions above. You may also include additional comments for the author, including concerns about dual publication, research ethics, or publication ethics. (Please upload your review as an attachment if it exceeds 20,000 characters)

Reviewer #1: This paper looks at a relatively under researched area for the context and would be a great addition to literature.

The methods however needs revisions. There are a few missing pieces that the authors must consider to be able to reach the conclusions that they arrived at. These have been pointed out in the attached reviewed manuscript and partly include consideration of factors that may have influenced the observed relationships. Some of these have been published from the apparently the same dataset by the same authors. For instance, the authors had concluded in a recent publication that the level of above-site oversight might influence the management outcomes observed in the same facilities.

It is also not clear why a clustering was done when individual managerial performance scores were available as well as individual personality trait scores.

There are other minor revisions that the authors are invited to consider in addition to the methodological concerns raised above. All these are in the attachment.

Looking forward to reviewing an updated analysis.

Reviewer #2: Reviewer Comments on the Manuscript

Overall Assessment

The manuscript explores an important and under-studied topic: the relationship between manager personality traits and managerial capacity in community-based HIV service facilities. The study contributes to the literature on health systems management in resource-limited settings. However, several conceptual, methodological, analytical, and reporting issues limit the strength of the conclusions.

Study Title

The title suggests managerial performance; however, the analysis appears to assess managerial capacity or adherence to management practices rather than performance. As such, the term ‘managerial performance’ may be misleading and should be reconsidered

Recommendation

• Consider revising the title to reflect the actual variable measured

Abstract

The abstract does not clearly specify the study design used

The statistical analysis sequence is not very clear. How was the transition from Cluster analysis to Logit regression made.

The abstract suggests: “personality traits can boost management capacity” but the study only shows associations, not causal relationships..

The statement: “stable personality consistently plays a positive and statistically significant role “is too general and not specific enough. Is there a statistical significance measure associated with this conclusion.

Recommendation

• The abstract should be strengthened by clearly defining the study design, clearly describing the key statistical analyses and the transition between them, and revising the conclusion to reflect association rather than causal relationships.

Introduction

While the introduction provides useful context on community-based HIV services, it requires stronger conceptual grounding. Specifically, it does not adequately explain based on existing literature or behavioral theories how personality traits may influence management performance in an HIV service delivery setting. The statement that personality traits in healthcare management have received ‘little attention so far’ (lines 90 – 93) should be further elaborated and supported.

Although the paper focuses on management capacity, its broader implications for HIV programme outcomes are not clearly articulated.

Recommendation

• The introduction if possible, should include a clearly defined conceptual model supported by relevant literature, a stronger theoretical explanation of how personality traits influence management performance, and a clearer articulation of the study’s relevance to health systems management, including how differences in management capacity relate to HIV programme outcomes.

Methods

General comment

The authors are encouraged to improve the clarity and organization of the methods section by structuring it under clear subheadings, including: Study Design, Study Setting, Sampling and Sample Size, measurement of variables, Data Collection Approaches, Data Analysis, and Ethical Considerations

Study Setting

The study setting had Limited explanation of Drop-In Centres

Recommendation

• The study setting section should provide a clearer and more detailed explanation of what Drop-In Centers are and their role in HIV service delivery for key populations, as the current description may not be sufficient for readers without prior experience in HIV programming or service delivery to fully understand their significance.

Sampling and Sample Size

Under the sampling and sample size section, the manuscript should provide stronger justification for the relatively small sample size, including how it was determined and whether it is sufficient to support the statistical analyses conducted. The authors should also discuss any constraints that limited sample expansion and the potential implications of the small sample size on the robustness and generalizability of the findings.

Data Collection Approaches

The study may be subject to self-reported survey bias, as managers assessed their own personality traits and management practices. This approach can introduce potential social desirability bias and self-evaluation bias, which may affect the accuracy and objectivity of the findings

Recommendation

• The authors should clarify what measures were implemented to minimize this bias

Measurement of Management Practices

While the World Management Survey framework was adapted, the aggregation procedure is not sufficiently described for readers unfamiliar with the method. In particular, the process used to derive and classify the management clusters lacks clarity and should be more explicitly explained.

Recommendation

• The authors should clearly describe the aggregation procedure and how management clusters were derived and classified to improve transparency and reproducibility.

Personality Trait Measurement

The manuscript does not specify which version of the Big Five Inventory was used (e.g., BFI-10, BFI-44), and it does not report the reliability of the personality scale. Also, the cultural validity of the instrument in the contexts of Kenya and Malawi has not been discussed.

Recommendation

• Specify the measurement tool by indicating which version of the Big Five Inventory was used, including appropriate references and justification for its selection.

• Address cultural validity by indicating the cultural appropriateness and validity of the instrument in the contexts of Kenya and Malawi, including any prior validation studies or adaptations made for these settings.

Results

The results section has several strengths, including clear presentation of cluster differences in tables and well-articulated comparisons of personality traits.

The authors should provide a clear explanation for running eight (8) models, in table 3 and 4 what goes into each model.

Recommendations

• The authors should provide a clear and detailed explanation of the modelling approach, including the rationale for running the eight models presented in Tables 3 and 4, and describe the variables and specifications included in each model

Discussion

General comment

The authors are encouraged to improve the structure of the manuscript by including clearly defined subsections following the discussion, specifically for Strengths, Limitations, and Recommendations, to enhance clarity and provide a balanced interpretation of the study findings.

Limitations

• The authors identified small sample size as a limitation, however, it would be important to clarify why the sample could not be expanded by including additional DICs, particularly given that the LINKAGES programme is implemented across multiple African countries. Providing justification for the sampling scope would help readers better understand the constraints and contextual factors influencing the study design.

**********

6. PLOS authors have the option to publish the peer review history of their article (what does this mean?). If published, this will include your full peer review and any attached files.

If you choose “no”, your identity will remain anonymous but your review may still be made public.

**Do you want your identity to be public for this peer review?**  For information about this choice, including consent withdrawal, please see our Privacy Policy.

Reviewer #1: **Yes:**  Edward Oladele

Reviewer #2: No

**********

[NOTE: If reviewer comments were submitted as an attachment file, they will be attached to this email and accessible via the submission site. Please log into your account, locate the manuscript record, and check for the action link "View Attachments". If this link does not appear, there are no attachment files.]

To ensure your figures meet our technical requirements, please review our figure guidelines: https://journals.plos.org/plosone/s/figures

You may also use PLOS’s free figure tool, NAAS, to help you prepare publication quality figures: https://journals.plos.org/plosone/s/figures#loc-tools-for-figure-preparation.

NAAS will assess whether your figures meet our technical requirements by comparing each figure against our figure specifications.

Attachment Submitted filename: PONE-D-26-01918_rev.pdf

Attachment Submitted filename: Reviewer Comments Updated.docx

# Author comment: Author response to Decision Letter 1

**DOI:** 10.1371/journal.pone.0352752.r002

27 May 2026

Reviewer:1

1. This paper looks at a relatively under researched area for the context and would be a great addition to literature. The methods however needs revisions. There are a few missing pieces that the authors must consider to be able to reach the conclusions that they arrived at. These have been pointed out in the attached reviewed manuscript and partly include consideration of factors that may have influenced the observed relationships. Some of these have been published from the apparently the same dataset by the same authors. For instance, the authors had concluded in a recent publication that the level of above-site oversight might influence the management outcomes observed in the same facilities.

Response: Thanks. Yes, previously it was found that organizational structure and oversight are relevant for management. In the light of that, in this paper we included the variable “IPs with 2+ DICs” as a control, which can be a proxy for oversight. This variable distinguishes between DICs that are part of a larger network (high potential for above-site oversight) versus those that are unique entities. By including this in the econometric models, we ensure that the reported effects of personality traits are independent of the influence of this oversight.

It is also worth noting that our results indicate that while oversight is important, managerial personality traits remain statistically significant predictors of a DIC being in the "high-management" cluster, even when these organizational factors are held constant. This suggests that personality traits add unique value to our understanding of facility performance beyond institutional design.

2. It is also not clear why a clustering was done when individual managerial performance scores were available as well as individual personality trait scores.

Response: We appreciate the opportunity to clarify why we used the cluster analysis method over a simpler way to dichotomize the dependent variable (for example, split the management score at some point of its distribution).

First, any split (p50, mean, p75, etc.) imposes a cut that may have no actual meaning, especially if the data is skewed. K-means is data-driven, minimizing within-cluster variance to find natural densities in the data. Using a clustering method, we identify functional profiles of management. This captures the complementarity between different management dimensions, which any distribution split would collapse into a single, linear, less meaningful binary variable.

Second, it is worth noting that the eight variables selected for clustering better capture managerial agency, whereas the broader management scores reflect procedural compliance. Clustering allows us to isolate the “agency/authority effect” within a DIC. This is conceptually more appropriate for our study, as personality traits (our independent variables) are more likely to manifest in managers who possess agency to make decisions, rather than those simply following standardized paperwork protocols.

Finally, this approach provides a robust internal validity check. The variables used for clustering were entirely separate from the variables used to construct the management scores. The fact that our clustering naturally resulted in groups with statistically significant differences in these management scores proves that we are predicting a coherent management profile rather than a hand-picked artefact of the distribution.

3. There are other minor revisions that the authors are invited to consider in addition to the methodological concerns raised above. All these are in the attachment.

• Comments included in the PDF file

Page Section Location in Text Reviewer Comment Response

10 Abstract Line 37 -- personality traits via the Big Five Inventory Edit for clarity. Edited to clarify

10 Abstract Line 46 -- 'Higher managerial capacity is a key catalyst for organizational performance' The manager belongs to a high managerial capacity organization? Not that the manager themselves have a high managerial capacity? Edited to clarify

10 Abstract Line 48 -- 'by fostering and improving relevant personality traits' This does not come from your study. Deleted

11 Introduction Line 58 -- services sentence (community-based facilities face significant challenges) Seems to be an abrupt sentence. Link to preceding sentence. Edited to connect

11 Introduction Line 61-62 -- 'Community-based facilities frequently operate under significant human resource constraints' This also needs to be connected to preceding concepts. Edited to connect

11 Introduction Line 70 -- 'A growing body of research highlights the importance of effective management in organizations' Effective management may be considered critical in all organizations. You may want to rephrase. Edited to clarify

12 Introduction Line 75 -- 'Effective management is rooted partly in the implementation of good management practices' Rooted in or evidenced by? Edited

12 Introduction Line 81-82 -- 'Research suggests that both management capacity and personality traits of facility managers [matter]' Any research suggesting this? Edited/Added references

13 Introduction Line 100 -- 'investigate the relationship between managerial performance and the Big Five personality traits' Reference? Added references

13 Introduction Line 105-106 -- 'By identifying the traits associated with managerial performance' Move to methods? Edited

13 Introduction Line 102 -- 'Stability -- as well as the higher-order traits (or meta-traits) of Plasticity and Stability' Separate traits or aggregate traits? Edited to clarify

14 Methods -- Data Collection Line 136 -- SurveyMonkey platform, 44 DIC managers contacted How were these selected? Clarification added

15 Methods -- Data Collection Line 138 -- 'two DICs in Kenya were overseen by the same manager' Did you analyze the same manager twice? Clarification added

16 Methods -- Management Domain Scores Line 162 -- 'detailed information in each management domain is provided in Table A1' S1? Changed

16 Methods -- Personality Traits Line 165 -- Anderson's (2008) procedure for normalizing items You may want to include a brief justification. Clarification added

17 Methods -- Cluster Analysis Line 195-196 -- k-means clustering explanation Why cluster? Since you had managerial performance for each facility and manager personality traits, was it possible to assess the managerial performance for each facility as per WMS and examine the influence of personality traits while controlling for confounders? Clarification added

19 Methods -- Logit Models Line 227-228 -- probability of being in the higher management cluster Each DIC had a measure of managerial performance, a manager and the personality trait of that manager but this approach first clusters the facilities and then modeled a probability that the facility would fall in a cluster given the manager's personality trait. Does that seem like a roundabout way of examining what you had straightforward data to do? Clarification added

19 Methods -- Logit Models Line 228 -- manager's formal education and accumulated experience Based on the context described, there are so many factors that could really influence any observed relationships. Did the managers get training on the job, how long were they on this job (not cumulative experience), did funding levels differ, what management support was available, was the intensity of support different from facility to facility? Response below

19 Results -- Table Table -- second set of observations (below the top half) Include a heading for this set of observations that are different from the top half. Included

20 Methods -- Logit Models Line 229 -- manager experience with key populations Were the managers at their jobs long enough to influence the managerial performance observed for their facilities? This is a critical missing piece. Response below

22 Results -- Discussion Line 282 -- Big Five results consistent with meta-trait results Do the authors expect these to not be consistent? Are the meta-personality traits aggregates of the individual traits? Addressed in the manuscript

23 Results -- Table Page 23 -- table figure/number 3 decimal spaces. Done

25 Discussion Line 315 -- 'funding sources' Were these not LINKAGES-funded facilities? Yes, edited accordingly.

26 Discussion Line 318-319 -- 'likely streamlining new information for DIC managers' This is an important factor to account for. Was it uniform across facilities regardless of performance? We do not know and we have mentioned this in in text.

26 Discussion Line 334 -- 'leadership development training and the design of support programs' Did the authors review the training package for the managers? Was there training? Was it devoid of recommended considerations. We do not have this information. However, here we are talking about community-based organizations generally, and we have clarified that.

27 Discussion Line 338 -- 'interventions should be tailored to individuals' age and personality' This was not explored at all in this study. Deleted

27 Limitations Line 345 -- 'effects Our findings come from...' Full stop missing. Added

28 Acknowledgements Line 366 -- 'FIH360' FHI 360. Corrected

28 Acknowledgements Line 369-370 -- USAID acknowledgement You may want to direct this to the US Government who provided the funding through USAID. Revised

• Based on the context described - there are so many factors that could really influence any observed relationships and the authors will do well to include the data in their analysis. Did the managers get training on the job, how long were they on this job (not cumulative experience), did funding levels differ even though all were supported by LINKAGES, what management support was available, was the intensity of support different from facility to facility? The authors have published some findings from this data set and may be able to layer in some of those confounders here.

Response: We appreciate the reviewer’s insight into the complex organizational factors that influence managerial capacity in these type of health organizations. While we acknowledge that variables such as job-specific training, tenure in the current role, and varying levels of IP support are theoretically relevant, we have intentionally limited the number of covariates in our econometric models to avoid overfitting and to preserve the statistical power of our analysis given the relatively small sample size. Adding further variables would significantly reduce our degrees of freedom and potentially lead to unreliable estimates. However, we believe these concerns are substantially mitigated by our existing model specifications. We included country fixed effects, capturing differences in national funding landscapes, Ministry of Health training protocols, and broader institutional support. This ensures that our results are identified through within-country variation, effectively controlling for systematic differences between the Kenyan and Malawian contexts. We also included IP-type, which proxy for the level of centralized administrative support and the standardized training regimes often associated with larger organizational structures. Finally, we have years of experience, which captures the accumulated human capital of the manager. By using these broader categorical controls, we maintain a parsimonious model that captures the latent influence of the factors mentioned by the reviewer without compromising the integrity of our statistical findings.

• Were the managers at their jobs long enough to influence the managerial performance observed for their facilities? This is a critical missing piece.

Response: We agree that the duration of a manager’s influence is an important consideration. While our data does not include a specific measure of tenure at the current facility, we believe this factor is effectively captured by our measure of years of experience. As shown in the regression results, this variable was a significant and robust predictor of belonging to the higher management cluster across all specifications. This suggests that the management practices we observed are a function of the manager’s accumulated expertise.

Reviewer:2

The manuscript explores an important and under-studied topic: the relationship between manager personality traits and managerial capacity in community-based HIV service facilities. The study contributes to the literature on health systems management in resource-limited settings. However, several conceptual, methodological, analytical, and reporting issues limit the strength of the conclusions.

1. Study Title

The title suggests managerial performance; however, the analysis appears to assess managerial capacity or adherence to management practices rather than performance. As such, the term ‘managerial performance’ may be misleading and should be reconsidered. Recommendation. Consider revising the title to reflect the actual variable measured.

Response: Thanks. The title of the manuscript is now: “Personality Traits and the Managerial Capacity of Community-Based Facilities Providing HIV Services to Key Populations in Kenya and Malawi”

2. Abstract

The abstract does not clearly specify the study design used. The statistical analysis sequence is not very clear. How was the transition from Cluster analysis to Logit regression made. The abstract suggests: “personality traits can boost management capacity” but the study only shows associations, not causal relationships. The statement: “stable personality consistently plays a positive and statistically significant role “is too general and not specific enough. Is there a statistical significance measure associated with this conclusion. Recommendation. The abstract should be strengthened by clearly defining the study design, clearly describing the key statistical analyses and the transition between them, and revising the conclusion to reflect association rather than causal relationships.

Response: We thank the reviewer for these suggestions. We have revised the abstract.

The study design is now stated clearly as a cross-sectional, quantitative analysis of 45 facilities providing HIV services to key populations in Kenya and Malawi. The analysis is now described in two stages. We first use k-means cluster analysis on management practice data to identify subgroups of facilities with similar management profiles. We then use cluster membership as the dependent variable in logit regression models, estimating the probability that a facility belongs to the higher-managerial-capacity cluster as a function of managers' personality traits, controlling for education, experience, and facility characteristics. We have revised the language throughout the abstract to reflect associations rather than causal relationships.

3. Introduction

While the introduction provides useful context on community-based HIV services, it requires stronger conceptual grounding. Specifically, it does not adequately explain based on existing literature or behavioral theories how personality traits may influence management performance in an HIV service delivery setting. The statement that personality traits in healthcare management have received ‘little attention so far’ (lines 90 – 93) should be further elaborated and supported. Although the paper focuses on management capacity, its broader implications for HIV programme outcomes are not clearly articulated. Recommendation. The introduction if possible, should include a clearly defined conceptual model supported by relevant literature, a stronger theoretical explanation of how personality traits influence management performance, and a clearer articulation of the study’s relevance to health systems management, including how differences in management capacity relate to HIV programme outcomes.

Response: We thank the reviewer for these suggestions and we have revised the introduction to address them.

We have added Trait Activation Theory (Tett & Burnett, 2003) as the theoretical framework for the personality–management relationship. The theory holds that personality traits translate into work behaviors when the demands of the role activate them. We apply it to community-based HIV service delivery by describing the demands managers face: coordinating comple

Attachment Submitted filename: RR_letter_D26_01918_v27May26.docx

# Peer review: Decision Letter 1

**DOI:** 10.1371/journal.pone.0352752.r003

**Authors:** Kwasi Torpey, Kwasi Torpey

15 Jun 2026

Personality Traits and the Managerial Capacity of Community-Based Facilities Providing HIV Services to Key Populations in Kenya and Malawi

PONE-D-26-01918R1

Dear Dr. Salas-Ortiz

We’re pleased to inform you that your manuscript has been judged scientifically suitable for publication and will be formally accepted for publication once it meets all outstanding technical requirements.

Within one week, you’ll receive an e-mail detailing the required amendments. When these have been addressed, you’ll receive a formal acceptance letter and your manuscript will be scheduled for publication.

An invoice will be generated when your article is formally accepted. Please note, if your institution has a publishing partnership with PLOS and your article meets the relevant criteria, all or part of your publication costs will be covered. Please make sure your user information is up-to-date by logging into Editorial Manager at Editorial Manager® and clicking the ‘Update My Information' link at the top of the page. For questions related to billing, please contact billing support.

If your institution or institutions have a press office, please notify them about your upcoming paper to help maximize its impact. If they’ll be preparing press materials, please inform our press team as soon as possible -- no later than 48 hours after receiving the formal acceptance. Your manuscript will remain under strict press embargo until 2 pm Eastern Time on the date of publication. For more information, please contact onepress@plos.org.

Kind regards,

Professor Kwasi Torpey, MD PhD MPH

Academic Editor

PLOS One

Additional Editor Comments (optional):

Thanks for the revisions. The authors have satisfactorily addressed then major issues raised in the initial review. The authors have clearly articulated the difference between this paper and an already published article that shows that above site oversight may influence management outcomes. The authors make clear distinction to demonstrate that the effect of personality traits is independent of the oversight effect. The rationale for the use of cluster analysis is provided as it facilitates capturing managerial agency while isolating its effect. The methodological details within the abstract and the main manuscript has been significantly improved with structured subheadings. The sampling, sample size, data collection approaches and measurement of management practices have been further clarified. Minor and editorial comments have been attended to

Overall, the revisions by the authors have improved the clarity of the manuscript to readers.

Reviewers' comments:

Reviewer's Responses to Questions

**Comments to the Author**

1. If the authors have adequately addressed your comments raised in a previous round of review and you feel that this manuscript is now acceptable for publication, you may indicate that here to bypass the “Comments to the Author” section, enter your conflict of interest statement in the “Confidential to Editor” section, and submit your "Accept" recommendation.

Reviewer #2: All comments have been addressed

**********

2. Is the manuscript technically sound, and do the data support the conclusions?

The manuscript must describe a technically sound piece of scientific research with data that supports the conclusions. Experiments must have been conducted rigorously, with appropriate controls, replication, and sample sizes. The conclusions must be drawn appropriately based on the data presented.

Reviewer #2: Yes

**********

3. Has the statistical analysis been performed appropriately and rigorously?

Reviewer #2: Yes

**********

4. Have the authors made all data underlying the findings in their manuscript fully available?

The PLOS Data policy requires authors to make all data underlying the findings described in their manuscript fully available without restriction, with rare exception (please refer to the Data Availability Statement in the manuscript PDF file). The data should be provided as part of the manuscript or its supporting information, or deposited to a public repository. For example, in addition to summary statistics, the data points behind means, medians and variance measures should be available. If there are restrictions on publicly sharing data—e.g. participant privacy or use of data from a third party—those must be specified.

Reviewer #2: Yes

**********

5. Is the manuscript presented in an intelligible fashion and written in standard English?

PLOS ONE does not copyedit accepted manuscripts, so the language in submitted articles must be clear, correct, and unambiguous. Any typographical or grammatical errors should be corrected at revision, so please note any specific errors here.

Reviewer #2: Yes

**********

6. Review Comments to the Author

Please use the space provided to explain your answers to the questions above. You may also include additional comments for the author, including concerns about dual publication, research ethics, or publication ethics. (Please upload your review as an attachment if it exceeds 20,000 characters)

Reviewer #2: The authors have responded appropriately to all comments and suggestions. I am satisfied with the revisions and recommend the manuscript for publication.

**********

7. PLOS authors have the option to publish the peer review history of their article (what does this mean?). If published, this will include your full peer review and any attached files.

If you choose “no”, your identity will remain anonymous but your review may still be made public.

**Do you want your identity to be public for this peer review?**  For information about this choice, including consent withdrawal, please see our Privacy Policy.

Reviewer #2: No

**********

# Editor report: Acceptance letter

**DOI:** 10.1371/journal.pone.0352752.r004

**Authors:** Kwasi Torpey, Kwasi Torpey

PONE-D-26-01918R1

PLOS One

Dear Dr. Salas-Ortiz,

I'm pleased to inform you that your manuscript has been deemed suitable for publication in PLOS One. Congratulations! Your manuscript is now being handed over to our production team.

At this stage, our production department will prepare your paper for publication. This includes ensuring the following:

* All references, tables, and figures are properly cited

* All relevant supporting information is included in the manuscript submission,

* There are no issues that prevent the paper from being properly typeset

You will receive further instructions from the production team, including instructions on how to review your proof when it is ready. Please keep in mind that we are working through a large volume of accepted articles, so please give us a few days to review your paper and let you know the next and final steps.

Lastly, if your institution or institutions have a press office, please let them know about your upcoming paper now to help maximize its impact. If they'll be preparing press materials, please inform our press team within the next 48 hours. Your manuscript will remain under strict press embargo until 2 pm Eastern Time on the date of publication. For more information, please contact onepress@plos.org.

You will receive an invoice from PLOS for your publication fee after your manuscript has reached the completed accept phase. If you receive an email requesting payment before acceptance or for any other service, this may be a phishing scheme. Learn how to identify phishing emails and protect your accounts at https://explore.plos.org/phishing.

If we can help with anything else, please email us at customercare@plos.org.

Thank you for submitting your work to PLOS ONE and supporting open access.

Kind regards,

PLOS ONE Editorial Office Staff

on behalf of

Professor Kwasi Torpey

Academic Editor

PLOS One
